# Establishment of a zebrafish inbred strain, M-AB, capable of regular breeding and genetic manipulation

**DOI:** 10.1038/s41598-024-57699-3

**Published:** 2024-03-29

**Authors:** Kenichiro Sadamitsu, Fabien Velilla, Minori Shinya, Makoto Kashima, Yukiko Imai, Toshihiro Kawasaki, Kenta Watai, Miho Hosaka, Hiromi Hirata, Noriyoshi Sakai

**Affiliations:** 1 College of Science and Engineering, Aoyama Gakuin University, Sagamihara, 252-5258 Japan; 2 Model Fish Genetics Laboratory, National Institute of Genetics, Mishima, 411-8540 Japan; 3 Department of Biology, Keio University, Yokohama, 223-8521 Japan; 4 Department of Genetics, SOKENDAI, Mishima, 411-8540 Japan; 5 Present Address: Faculty of Science, Toho University, Funabashi, 274-8510 Japan

**Keywords:** Inbred, Sib-pair mating, Strain, Whole-genome sequence, Zebrafish, Developmental biology, Genetics

## Abstract

Inbred strains of organisms are genetically highly uniform and thus useful for life science research. We have previously reported the ongoing generation of the zebrafish IM strain from the India (IND) strain through full sib-pair mating for 16 generations. However, the IM fish laid a small number of offspring and had a short lifespan, implying the need for discreet care in breeding. Here, we report the subsequent establishment of IM strain as well as the generation of a new inbred zebrafish strain, Mishima-AB (M-AB). M-AB was derived from the *AB strain by full sib-pair mating for over 20 generations, which fulfills the general criterion for the establishment of an inbred strain. In contrast to the IM case, maintenance of the M-AB strain by sib-pair mating required almost no special handling. Genome sequencing of IM individuals from the 47th generation and M-AB individuals from the 27th generation revealed that SNP-based genomic heterogeneity across whole-genome nucleotides was 0.008% and 0.011%, respectively. These percentages were much lower than those of the parental IND (0.197%) and *AB (0.086%) strains. These results indicate that the genomes of these inbred strains were highly homogenous. We also demonstrated the successful microinjection of antisense morpholinos, CRISPR/Cas9, and foreign genes into M-AB embryos at the 1-cell stage. Overall, we report the establishment of a zebrafish inbred strain, M-AB, which is capable of regular breeding and genetic manipulation. This strain will be useful for the analysis of genetically susceptible phenotypes such as behaviors, microbiome features and drug susceptibility.

## Introduction

The genetic background of several organisms has been shown to affect phenotypes associated with genetic variants or experimental manipulation^1–3^. Since genetically uniform animal strains eliminate individual differences in genetic background, they are useful for analyzing biological responses with high individual differences, such as behaviors, microbiome features and drug susceptibility. In several vertebrate animal species, such as mice, rats, and medakas, all of which produce offspring with an equal female-to-male ratio by a sex chromosome, the descendants from a single pair through full sib-pair mating have been produced for 20 or more consecutive generations to establish an inbred strain^4–6^. By the 20th generation of full sib-pair mating, allelic heterogeneity of the genome is expected to be less than 1.4%^7^. Although zebrafish have been recognized as a major model organism for studying biological processes in live vertebrate animals, inbred strains of zebrafish have not been successfully generated by consecutive sib-pair mating, probably due to an imbalanced sex ratio and the production of fragile offspring, with the latter being referred to as inbreeding depression. Parthenogenesis has been used to generate a zebrafish strain with low allelic heterogeneity^8,9^. However, the heterogeneities of the C32 and SJD strains, both of which have been produced by parthenogenetic generations, were 11% and 7% to 2120 SNP loci, respectively^10^.

We have previously reported the generation of a zebrafish IM strain, which was derived from the heterogeneous wild-type India (IND) strain through consecutive sib-pair mating for 16 generations^11^. After publishing this initial report, we continued full sib-pair mating for over 20 generations, which is the standard criterion for the establishment of an inbred strain in mice and rats^5,6^. The repetitive sib-pair mating of the IM strain was further continued to reach 50 generations in 2023. However, the reproductive period of this strain fish is short, and it currently spawns a small number of eggs. Since breeding of the IM strain fish requires careful handling, the establishment of a separate inbred strain that can be maintained by regular care has been a desire of the research community.

In this study, we attempted to generate a new inbred strain that is better at breeding than the IM strain. To eliminate unwanted genetic variants that cause inbreeding depression, we employed the *AB strain, which was generated after efforts were made to remove harmful variants through the selection of good homozygous gynogenetic females that produce haploid embryos showing normal early embryogenesis^12^. We carried out full sib-pair mating for more than 20 generations and established an inbred strain named Mishima-AB (M-AB). Genome sequencing of IM and M-AB individuals revealed that the genetic heterogeneities of these strains were low. Embryos of the M-AB strain fish were robust in development, and applicable to microinjections at the 1-cell stage. Collectively, the inbred M-AB strain is amenable for breeding and useful for genetic studies in zebrafish.

## Results

### Subsequent establishment of the inbred IM strain

The IM strain was generated by repetitive sib-pair mating of the IND strain for 16 generations^11^. We continued sib-pair mating for a total of 20 generations and here declare the establishment of the IM strain as an inbred strain (Fig. S1A). Further sib-pair mating of the IM strain fish was performed, reaching the 50th generation in 2023 (Fig. S1B–D). During this sib-pair mating, inbreeding depression was observed in many branches. The embryonic lethal phenotype with a small head, an underdeveloped lower jaw and poor circulation, which we have previously referred to as the Tübingen-Mishima (TM) phenotype^11^, was observed in the clutches of the I_16_-3, I_20_-5, I_22_-9, I_23_-4, I_23_-5 and I_23_-10 pairs (Table S1). The total number of eggs per clutch in generation 20 was less than half of that in generation 0 (Fig. S2A). The efficiency of fertilization (number of fertilized eggs/number of total eggs) declined to 50% in the 19th generation (Fig. S2B,C). The survival rate of offspring at 3 days post fertilization (dpf) also declined after the 14th generation (Fig. S2D,E). Taking into account these inbreeding depression data, we generated various branches of the IM subfamily after the 15th generation and selected a few good branches for subsequent maintenance. This labor-intensive inbreeding improved the number of eggs, efficiency of fertilization and survival of offspring from the 23rd generation. The 50th generation of the IM strain is currently maintained but has a low fertility rate and a small number of embryos (Table S2).

### Generation of the inbred M-AB strain

**Figure 1:**
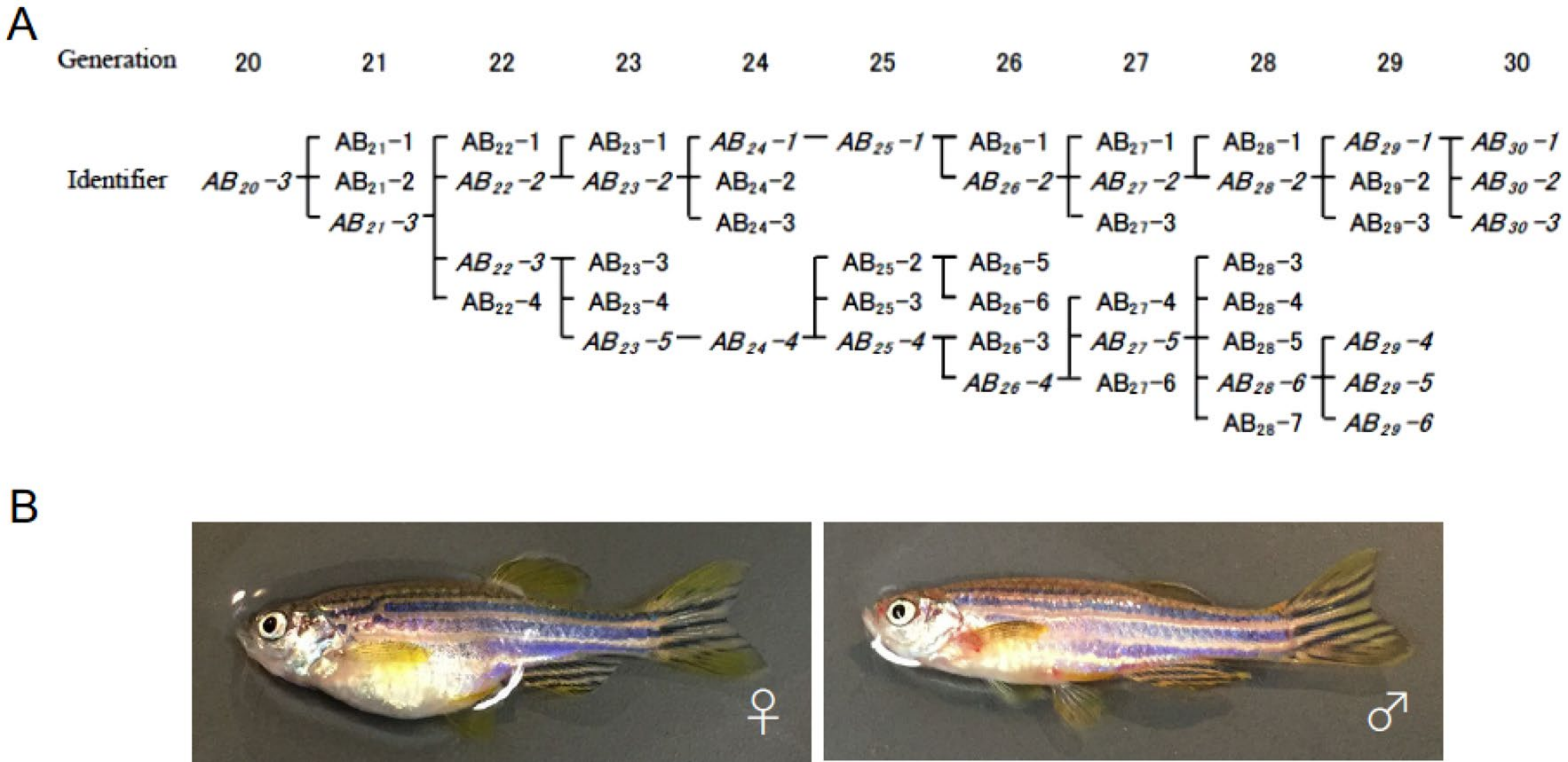
Pedigree of the M-AB strain. (**A**) Pedigree of the M-AB strain from generation 20 to 30. Pairs in italics indicate pairs that are connected to existing pairs. (**B**) Female and male fish of the M-AB strain.

To attempt to generate a new inbred line with lower inbreeding depression, we employed the *AB strain, which was generated from the AB strain by selecting good parthenogenesis-derived females^12^. A course of sib-pair mating started in 2010 followed almost the same procedure as that used for the IM strain except for maintaining a smaller number of branches (Fig. S3A). During consecutive inbreeding, we neither observed the fragile TM phenotypes nor found a reduction in egg number or fertility rate. However, the *AB-derived strain fish often gave rise to male-biased progeny that resulted in the discontinuation of several branches, i.e., AB_19_-7 and AB_19_-10 pairs (Fig. S3B). This tendency of male bias was mitigated by switching the larval diet from paramecia to rotifers and by lowering the growth density of larvae and juveniles in a tank (see “Materials and methods”). Sib-pair mating was continued for over 20 generations, and we declare the establishment of the M-AB strain as an inbred strain. Consecutive sib-pair mating of the M-AB strain reached the 30th generation in 2023 (Fig. 1A).

The M-AB fish looked healthy (Fig. 1B) and were maintained by regular fish care but with a concern of a male-biased tendency that appeared in several generations. The number of fertilized eggs (~ 200 embryos), the efficiency of fertilization (~ 90%) and the length of the body at 30 dpf were almost comparable between M-AB in the 27th generation and the parental *AB strain (Fig. S4A–D).

### Phylogenetic analysis of the M-AB and IM strains

**Figure 2:**
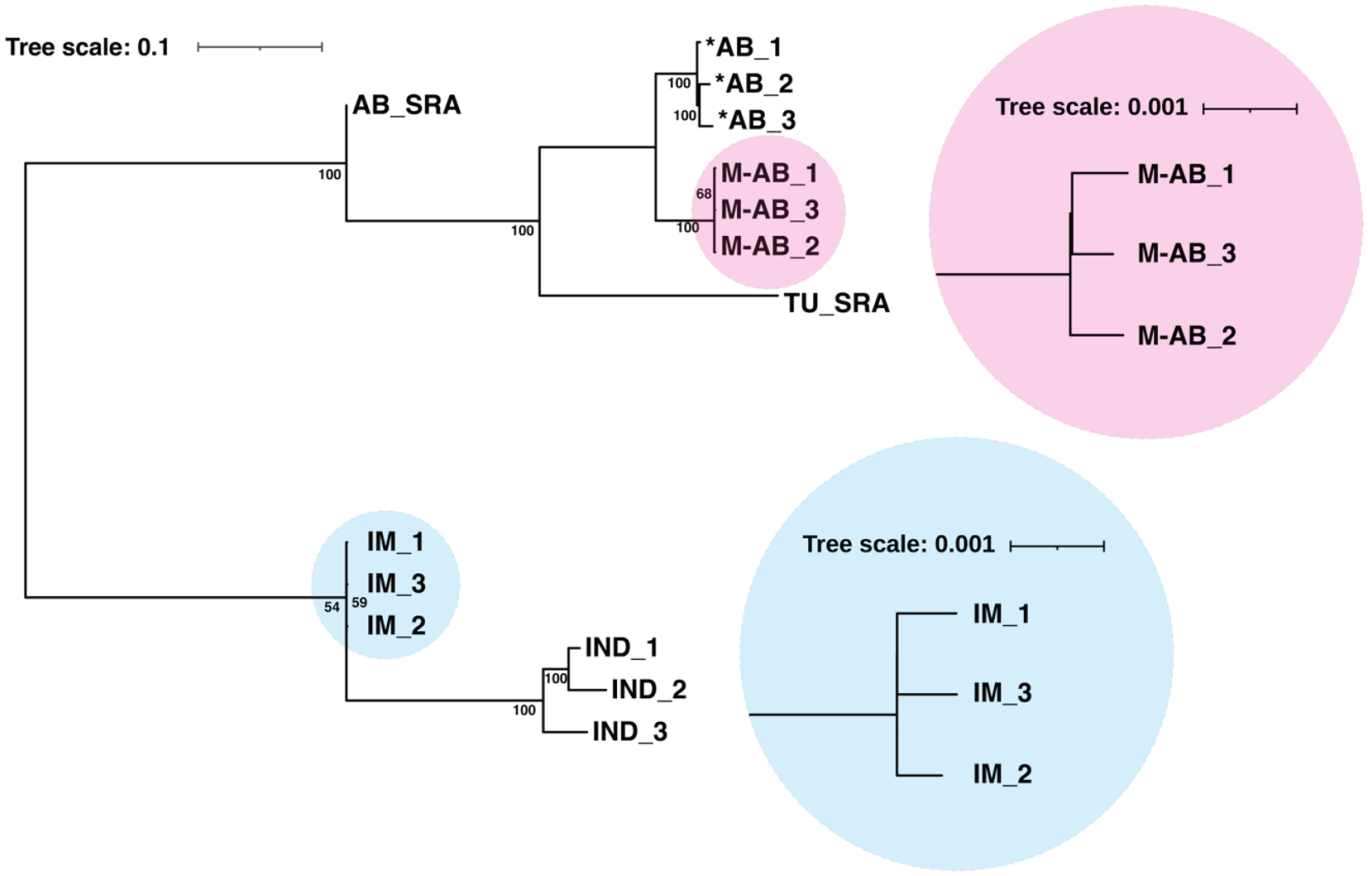
Phylogenetic tree of the M-AB and IM strains. Phylogenetic relationships of the M-AB, *AB, IM and IND strains with open data for AB_SRA and TU_SRA. This tree used 10,000 bootstrap replicates. Coefficients represent bootstrap values for tree nodes. Note that inbred strains have shorter branches than the other strains. The length of the branch indicates the diversity of that strain.

To assess the phylogenetic relationship of the inbred M-AB and IM strains with their parental strains, we performed whole-genome sequencing (WGS) of three individual fish of the 27th generation of M-AB (AB_27_-4), the 47th generation of IM (I_47_-2) and our lab stocks of *AB and IND. We also obtained genomic information for AB (AB_SRA) and TU (TU_SRA) from the NCBI Sequence Read Archive (SRA) as well as the zebrafish genome assembly of the TU strain (TU_GRCz11) from the Genome Reference Consortium^13^. We used TU_GRCz11 as a genome reference to identify SNPs in our 12 fish and the 2 archived sequences (Table 1). The total number of SNP positions found in the 14 total genomes was 24,312,666 (18.1% of the zebrafish genome). As expected, the number of SNPs found in the TU_SRA genome was lower than that in the other genome. Based on these SNPs, we created a phylogenetic tree of the 14 genomes by the maximum likelihood method using the standard transversional model^14,15^. The 3 individual fish of each strain were highly similar in each subgroup (Fig. 2). The M-AB and IM subgroups showed the closest relationship with their parental *AB and IND strains, respectively. These data indicate that M-AB and IM were phylogenetically isolated from their parentals by repetitive sib-pair mating, recapitulating the establishment of the inbred strains. We also noticed that TU_SRA rather than AB_SRA was phylogenetically close to M-AB and *AB, potentially suggesting that AB fish have highly diverged by long-term maintenance in institutions.

**Table 1:** Number of SNPs in each fish.

Strain	Number of SNPs
AB_SRA	10,370,466
TU_SRA	4,387,730
*AB_1	7,191,673
*AB_2	7,427,564
*AB_3	7,448,163
IND_1	11,159,260
IND_2	11,123,270
IND_3	10,981,208
M-AB_1	7,212,831
M-AB_2	7,060,857
M-AB_3	7,016,435
IM_1	9,298,861
IM_2	9,474,191
IM_3	9,231,903

### Genomic characteristics of the M-AB and IM strains

**Figure 3:**
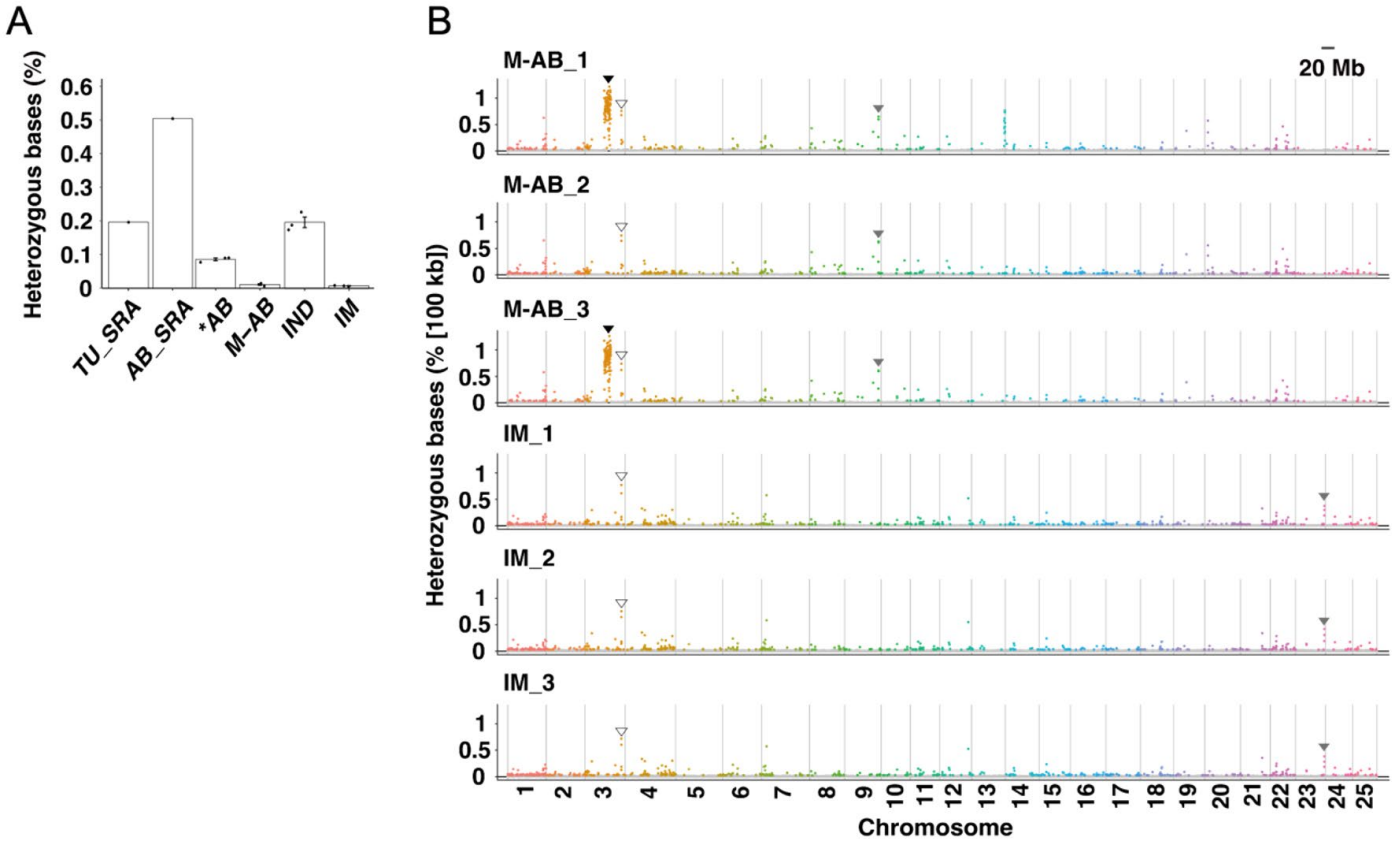
Genomic characteristics of the M-AB and IM strains. (**A**) The percentages of heterozygous nucleotides (number of heterozygous nucleotides/number of nucleotides in whole genome). Error bars represent the mean ± sem. Plots in the bar graph represent biological replicates. (**B**) The percentage of heterozygous SNPs in every 100 kb region from chromosomes 1 to 25 is indicated for three M-AB and three IM fish. Areas of interest are marked by triangles. Closed triangles: heterozygous region in two M-AB fish but not in the other M-AB individual; open triangles: heterozygous region common to all M-AB and IM fish, gray triangles: heterozygous region common to only M-AB fish or only IM fish. Detailed count data are available in Table S3.

To address the genomic features of the M-AB and IM strains, we evaluated the genomic homogeneity and heterogeneity of the inbred fish. Homogeneity was defined by the ratio of homozygous nucleotides (number of homozygous nucleotides/number of nucleotides in whole genome of 1.345 GB). The heterogeneity of the M-AB (0.011 ± 0.002%, n = 3) and IM (0.008 ± 0.001%, n = 3) strains was markedly lower than those of the parental *AB (0.086 ± 0.004%, n = 3) and IND (0.197 ± 0.016%, n = 3) strains as well as those of the AB_SRA (0.504%) and TU_SRA (0.196%) strains (Fig. 3A). Since heterogeneities of zebrafish strains were reported based on the total SNP numbers (number of heterozygous nucleotides/number of SNPs found in the analysis) in a previous study^8^, we also calculated the heterogeneity of each strain for the 24,312,666 SNP positions in a total of 14 genomes. The heterogeneity was 0.628 ± 0.129% in the M-AB strain and 0.469 ± 0.042% in the IM strain, which were also markedly lower than those of the *AB (4.770 ± 0.233%), IND (10.937 ± 0.876%), AB_SRA (27.894%) and TU_SRA (10.797%) strains. These data indicate that repetitive sib-pair mating significantly increased the allelic uniformity of the genome in both the M-AB and IM strains.

Although we analyzed the 27th generation of M-AB and the 47th generation of IM, allelically heterozygous nucleotides remained. To explore whether these “heterozygous nucleotides” are distributed evenly throughout the genome or accumulate at specific loci, we counted the number of heterogeneous nucleotides for every 100 kb in each chromosome (Fig. 3B; Table S3). Intriguingly, a prominent hot spot of heterozygous nucleotides was found at the middle of chromosome 3 in two M-AB individuals but not in the other M-AB_2 fish. Since the M-AB_2 fish that carries this region at homozygous survived to adulthood, the heterogeneity in this region would be removed by further sib-pair mating. We also found several genomic loci where heterogeneity was maintained in three M-AB fish (i.e., at the distal end in chromosome 9), three IM fish (i.e., at the distal end in chromosome 23) or all 6 fish (i.e., at the distal end in chromosome 3). These regions might contain closely located harmful genetic variants that are heterozygous for each different chromosomal allele.

### SNPs potentially affecting gene products in the M-AB and IM strains

**Figure 4:**
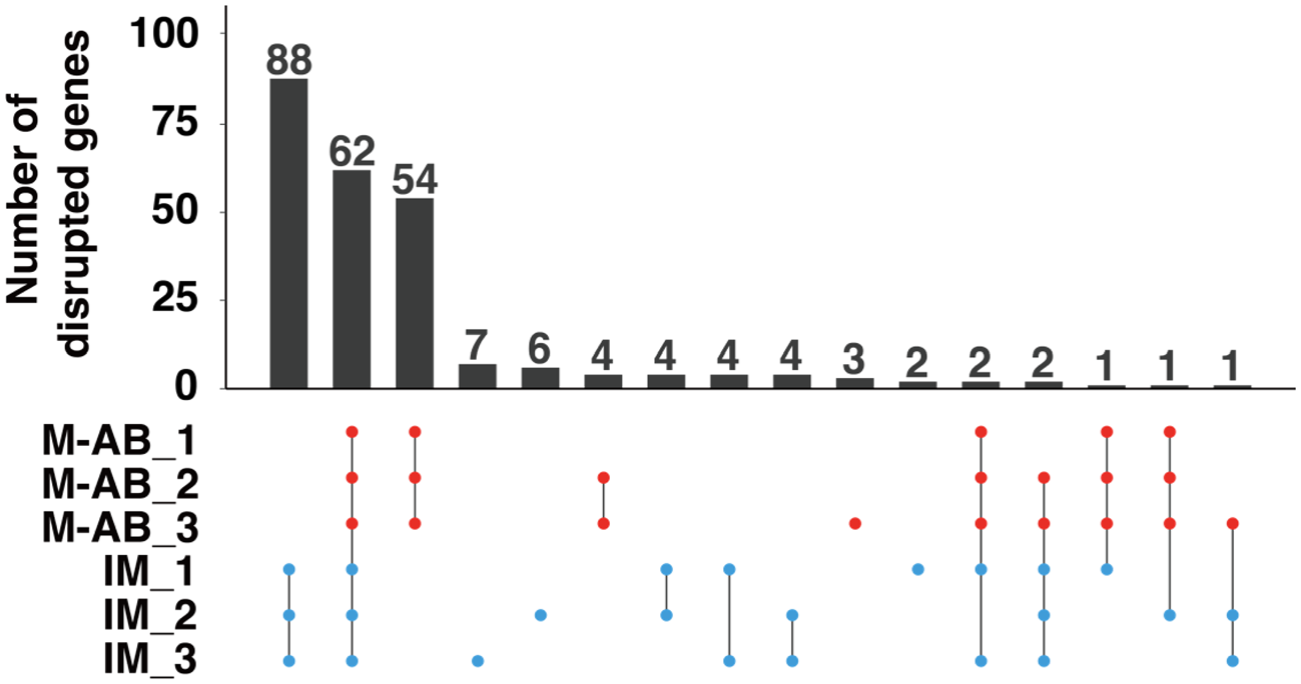
Number of genes potentially disrupted in the M-AB and IM strains. The homozygous SNPs that create nonsense codons or disrupt initiation codons, termination codons or 2-bp splicing junctions within protein coding genes were evaluated as gene disrupting SNPs. The upset plots indicate the overlap of disrupted genes in three M-AB and three IM fish.

Our WGS of M-AB and IM fish identified a number of SNPs that differed from the zebrafish genome assembly GRCz11. These SNPs were classified into heterozygous and homozygous SNPs in each fish. Generally, repetitive sib-pair mating promotes the accumulation of “homozygous SNPs” that affect the function of the gene products. Such homozygous SNPs represent genomic characteristics of a strain if the SNPs were commonly found in that strain. To evaluate the effects of these homozygous SNPs, we classified the SNPs into 6 groups by location with regard to exon‒intron regions and protein coding sequences (Table 2). In both the M-AB and IM strains, 96.9% of homozygous SNPs were found in either intron or intergenic regions, while 1.4% of homozygous SNPs were found in the 5′-UTR or 3′-UTR. The remaining 1.7% were located in the coding region or 2-bp splicing junctions. We then focused on homozygous SNPs that potentially disrupt genes by creating nonsense codons or disrupting initiation codons, termination codons or 2-bp splicing junctions. The overlap of homozygous SNPs that potentially disrupt genes between individuals revealed that most of the gene disruption was commonly found in three fish of each strain (Fig. 4). The list of the disrupted genes provided genetic features of the M-AB and IM strains (Table S4–6). For example, disrupted genes in the IM strain contained several genes relevant for reproduction, such as *cep72*^16^, *cxxc1*^17^, *cyp1a1*^18^, *fbxo42*^19^, *gsto2*^20^, *hsf5*^21^, *pex10*^22^, *psat1*^23^, and *rad51c*^24^. Among them, gene-disrupting SNPs in *cyp1a1* (Fig. S5) and *psat1* (Fig. S6) generated nonsense codons closer to the C-terminus of the coding sequences and thus might not affect the function of the gene products. However, the other gene-disrupting SNPs potentially affect the function of the gene products (Figs. S7–13).

**Table 2:** Number and ratio of homozygous SNPs.

	Protein-coding regions	2-bp Splicing junctions	5′-UTR	3′-UTR	Intron	Intergenic	Total
GRCz11.109 Percentage (Nucleotides)	5.556 (74,733,858)	0.089 (1,191,760)	0.434 (5,833,706)	1.695 (22,796,785)	65.838 (885,588,144)	26.389 (354,957,578)	100 (1,345,101,831)
M-AB (n = 3) Percentage (SNPs)	1.724 ± 0.007 (244,731)	0.004 ± 0.000 (538)	0.260 ± 0.001 (77,807)	1.122 ± 0.004 (159,280)	56.418 ± 0.010 (8,010,373)	40.473 ± 0.012 (5,746,573)	100 (14,239,303)
*AB (n = 3) Percentage (SNPs)	1.737 ± 0.007 (219,190)	0.004 ± 0.000 (477)	0.264 ± 0.001 (33,365)	1.125 ± 0.006 (141,900)	56.179 ± 0.065 (7,089,913)	40.691 ± 0.071 (5,135,388)	100 (12,620,234)
IM (n = 3) Percentage (SNPs)	1.760 ± 0.009 (330,730)	0.004 ± 0.000 (703)	0.264 ± 0.001 (49,533)	1.122 ± 0.003 (210,713)	56.277 ± 0.016 (10,571,984)	40.573 ± 0.021 (7,622,058)	100 (18,785,741)
IND (n = 3) Percentage (SNPs)	1.690 ± 0.013 (292,581)	0.004 ± 0.000 (648)	0.254 ± 0.002 (44,007)	1.103 ± 0.006 (190,859)	56.362 ± 0.073 (9,756,152)	40.586 ± 0.079 (7,025,722)	100 (17,309,968)

### Morpholino knockdown, CRISPR knockout and Tol2 transgenesis are possible in embryos of the M-AB strain

**Figure 5:**
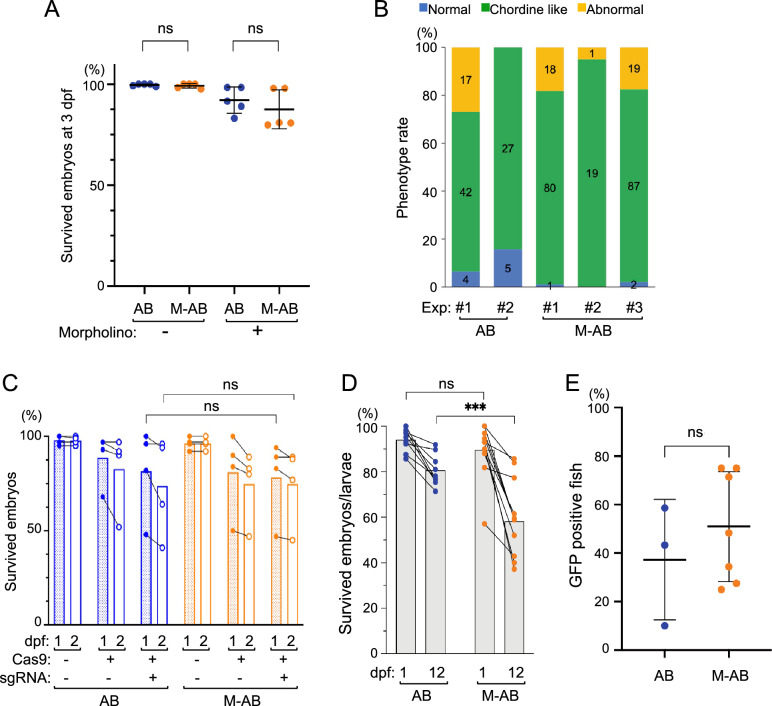
Survival of M-AB embryos was hardly affected by microinjections. (**A**) Survival of *chordin* MO-injected M-AB and *AB embryos (n = 5). (**B**) Approximately 80% of* chordin* MO-injected embryos showed *chordin*-deficient phenotypes. The numbers in the color bars indicate the number of samples. (**C**) Survival of M-AB and *AB embryos injected with *sqstm1* sgRNA and/or Cas9 protein (n = 4). (**D**) Survival of M-AB and *AB embryos/larvae injected with *ef1α:EGFP* plasmid and *tol2* mRNA (n = 9). Plots connected by lines indicate the same experimental group. (**E**) Percentages of fish (30 dpf) that harbor GFP-transgene detectable by PCR (*AB: n = 3, M-AB: n = 7). Error bars indicate the standard deviation.

Since the M-AB strain fish appears to be useful with fertilization and survival rates comparable to those of the parental *AB strain, we finally tested whether M-AB embryos can be genetically manipulated by microinjection of morpholino oligonucleotides (MOs), CRISPR/Cas9 and foreign genes. MOs against the *chordin* gene, which is involved in dorsoventral patterning in embryonic development, were injected into 1-cell embryos of M-AB and *AB clutches and subjected to survival and morphological analysis. The survival rate of MO-injected M-AB embryos (87.5 ± 9.6%) at 3 dpf was comparable to that of MO-injected *AB embryos (92.1 ± 6.5%) (Fig. 5A). More than 80% of MO-injected embryos displayed typical *chordin*-deficient phenotypes, such as expanded blood islands, U-shaped somites and short tail fins with multiple folds^25^ in both the M-AB and *AB backgrounds (Fig. 5B).

To attempt genome editing, we next injected CRISPR single-guide RNA (sgRNA) for the *sqstm1* gene^26^ and Cas9 protein into 1-cell embryos of the M-AB and *AB strains. The survival rates of injected embryos were comparable between the M-AB (74.8 ± 20.6%) and *AB (73.8 ± 26.3%) strains at 2 dpf (Fig. 5C). Our heteroduplex mobility assay suggested that insertions and/or deletions were generated at the target site of the *sqstm1* gene in all tested embryos of M-AB and *AB clutches (Fig. S14).

Finally, we injected the *ef1α:EGFP* plasmid with *tol2* mRNA into 1-cell embryos of the M-AB and *AB strains to test transposon-mediated transgenesis^27^. Although the survival rate of injected M-AB embryos (58.2 ± 17.0%) was slightly lower than that of injected *AB embryos (80.6 ± 6.8%) at 12 dpf, the survival rate of M-AB was more than 50% (Fig. 5D). The GFP gene was detected comparably in surviving M-AB embryos (50.9 ± 22.7%) and surviving *AB embryos (37.3 ± 24.9%) by PCR (Fig. 5E). After raising those embryos to the adult stage, we crossed them with wild-type fish and obtained embryos carrying the *ef1a:EGFP* transgene at 26.0% in the M-AB clutch and at 38.1% in the *AB clutch. Fluorescence detection of GFP was ascertained in 7.1% (9/127) of embryos obtained from the mating of three pairs of injected M-AB fish. These results suggest that the M-AB embryos are applicable to microinjection at the 1-cell stage and thus that M-AB is a useful inbred strain for the analysis of gene function.

## Discussion

The present study describes two genetically uniform zebrafish strains generated by consecutive full sib-pair mating for more than 20 generations. Since this criterion is used as a definition of an inbred strain in mice, rats and medakas, we can declare the establishment of IM and M-AB as inbred strains in zebrafish. With subsequent sib-pair mating, inbreeding of the IM strain has recently reached the 50th generation. Although we have provided IM embryos from the 20th–40th generations to the other labs, the IM strain is hardly maintained out of our lab, probably because IM fish lay only a small number of offspring during a short reproductive period. On the other hand, the M-AB strain, which was originated from *AB, did not show such a reproduction insufficiency. We have distributed M-AB embryos at the 26th–28th generations to several labs, and M-AB fish have been successfully maintained over the generation. Since *AB was derived from selected gynogenetic females of the AB strain^12^, genetic variants that adversely affect development and reproduction might have been removed from *AB. In accordance with the elimination of harmful variants, the embryonic lethal TM phenotype, which we often observed in IM offspring and TU-derived sib-pair progeny^11^, was not observed in M-AB embryos. The M-AB strain can be maintained by regular care and thus will be in demand in the zebrafish research community.

Sex is determined by the sex chromosome in mice, rats and medakas, whereas it is determined by both genetic and environmental factors in zebrafish^28^. Despite enormous efforts in sex determination studies, genetic factors that determine sex in zebrafish have not been unveiled. Interestingly, M-AB offspring have a tendency to become male in our breeding. Similar significant male bias has also been observed in the IM strain and in a previous sib-pair mating started from the WIK strain and wild Bangladesh fish^11,29^. Thus, inbred breeding likely promotes male bias in zebrafish. Adjustment of environmental factors enables changes in the sex ratio in zebrafish progeny. In our case, the tendency of male bias in the M-AB offspring was reduced by changing the larval diet from paramecia to rotifers and by decreasing the number of growing larvae and juveniles in a tank. Since the M-AB strain carries possible genetic factors for male bias but produces both females and males, it will also be useful to study genetic and environmental factors in sex determination.

Our previous genomic analysis of the IM strain using 100 SSLP markers suggested that heterogeneity of the IM strain from the 14th generation was 5%^11^. The current study using 24,312,666 SNPs, which we found as a difference from the zebrafish genome assembly, revealed that heterogeneities of the IM strain from the 47th generation and the M-AB strain from the 27th generation were 0.469% and 0.628%, respectively. Apart from these inbred lines, the heterogeneities of AB and TU were 27.894% and 10.797%, respectively. Our study was consistent with a former study using 2120 SNPs, in which the heterogeneities of AB and TU were calculated as 24.8% and 14.6%, respectively^10^. These data lead to the conclusion that the two inbred strains IM and M-AB, which were generated through repetitive sib-pair mating, were genetically highly homozygous.

The zebrafish genome assembly GRCz11 was generated by sequencing the TU genome. Based on this genome assembly, gene information, including transcript isoforms and protein coding sequences, was annotated in the Ensembl database. Our WGS of the IM identified a number of homozygous SNPs, which were shared in three IM fish, showing specific characteristics of the IM strain. The Ensembl suggested that some homozygous SNPs in the IM create nonsense codons or disrupt initiation codons, termination codons or splicing junctions, thereby leading to gene disruption. Such potentially disrupted genes included reproduction-related genes reported in zebrafish or in other vertebrates. For instance, a SNP in the *hsf5* gene, which is needed for spermatogenesis in zebrafish^21^, created a nonsense codon at the 357th amino acid residue (Fig. S7). Similarly, SNPs in the *cxxc1* and *rad51c* genes, both of which are essential for meiosis in mice^17,24^, resulted in protein truncation in the middle and first halves of the gene products, respectively (Figs. S8, S9). Other SNPs also suggested the disruption of *cep72*^16^, *fbxo42*^19^, *gsto2*^20^, and *pex10*^22^, which are relevant for reproduction. Such gene-disrupting SNPs may account for inbreeding depression, such as the low fertility rate of the IM strain. However, we also have to keep in mind that some annotations of genes in Ensembl comprise intron-containing transcripts and thus wrong protein coding sequences due to annotation errors^30^. Some cases of gene disruption retrieved from such homozygous SNPs may be false disruptions. Future studies of loss-of-function of those disrupted genes will elucidate the actual genetic causes of the reproduction-insufficient features of the IM strain.

On the other hand, we could not find homozygous SNPs that affect reproduction-related genes in the M-AB strain. A SNP that disrupts the termination codon of the reproduction-related gene *cep128*^31^ was found in all three M-AB fish and three IM fish (Table S6). However, it is unclear whether this SNP disrupts Cep128 function for the following reasons: the transcript of *cep128* (ENSDART00000165489.2) is incomplete, as it lacks a start codon, suggesting the presence of other splicing variants. The M-AB strain is maintained by regular care, and the M-AB embryos are applicable to microinjection for various purposes. Since M-AB is genetically highly uniform, this inbred strain will be useful, especially for the study of behaviors, microbiomes and drug susceptibility, which are often affected by individual genetic differences. Both the IM and M-AB strains are available from the National BioResource Project, which is a zebrafish stock center in Japan (https://nbrp.jp/en/resource/zebrafish-en/).

## Materials and methods

### Animals

The zebrafish *AB strain was kindly provided by Prof. Uwe Strähle in 2010. The IND strain fish was kindly provided by Dr. Yasuyuki Kishimoto. Both the *AB and IND strains were maintained by mass mating of 6 females and 8 males. The heterogeneous fish used in the analyses were bred under the same conditions as the M-AB strain.

Continued inbreeding of the IM strain and establishment of the M-AB strain were performed by basically the same procedure as described previously^11^. To reduce the time for maintenance, some modifications were made after the 28th generation of the IM strain and for the M-AB strain. First, the mating pair that did not produce a large number of offspring was not used for inbreeding. Second, the numbers of eggs and embryos were not exactly counted. Finally, the number of branches for inbreeding was not always five, especially when a sufficient number of offspring was obtained. The best mating pair was determined by subjective impressions of the offspring, including hardness of chorions and morphological normalcy up to 4 dpf. The embryos were raised at less than 50–100 embryos per 150 mm dish with replacement of approximately 1/5 of the system water every day. Feeding rotifers was started at 4 dpf, and brine shrimp were started at ~ 8 dpf, followed by observation of food eating. Five days after starting to eat brine shrimp, they were transferred into the circulation system (~ 20 larvae per 2 L tank). As M.S. has moved to Keio University in 2014, the M-AB strain after the 11th generation has been maintained as a substrain at two places: the National Institute of Genetics and Keio University.

To measure body size, zebrafish were anesthetized in 0.01% ethyl 4-aminobenzoate (w/v), and the body length and standard length were measured using a Vernier caliper.

### MO injection

For microinjection of antisense MOs, 2.3 nL of 0.05 mM *chordin* MOs or standard control MOs (GeneTools, Philomath, OR, USA) were injected into 1-cell stage embryos. Phenotype and survival were evaluated at 2 and 3 dpf, respectively.

### Genome editing

CRISPR/Cas9-mediated genome editing was performed based on published protocols^32,33^. Template DNA for sgRNA synthesis was prepared by amplification with a primer specific to *sqstm1*, a universal reverse primer and T4 DNA polymerase. After purification of the template DNA, sgRNA (taatacgactcactataGGACGGGTGTGATGGGCCGGgttttagagctagaa) was transcribed using a MEGAscript T7 Transcription Kit (Ambion, Austin, TX, USA) and purified with a MEGAclear Transcription Clean-up Kit (Ambion). The M-AB and *AB embryos were injected at the 1-cell stage with 2.3 nL of a mixture of 5 pmol/μL Cas9 Nuclease NLS Protein (Applied Biological Materials, Richmond, Canada) and 50 ng/μL *sqstm1* sgRNA. We evaluated the mutation at 1 dpf by heteroduplex mobility assay by PCR using the following primers^34^. Forward: 5′-GTCATATGGGTCCATCTCCAAT-3′; Reverse: 5′-AGTGCTATTCACCTCAAACACG-3′. Amplified PCR products were separated by polyacrylamide gel electrophoresis.

### *Tol2*-mediated transgenesis

To generate transgenic lines, microinjection was performed in 1-cell stage embryos with 2.3 nL of injection solution containing 15 ng/μL pTG-*egfp*^35^ and 25 ng/μL *tol2 transposase* mRNA^27^. Injected embryos were raised to the adult stage and crossed with the wild-type strain to generate F_1_ progeny. The expression of EGFP was visually assayed at 7 dpf. Transgenesis was confirmed by PCR using the genome extracted from the caudal fin crip of F0 fish at ~ 30 dpf and the F1 embryo at 1–4 dpf. The following primers were used to amplify the EGFP gene. 5′-ACCACATGAAGCAGCACGACT-3′; 5′-CTTCTGGTTGGGTCTTTGC-3′.

### Genome extraction

Fish were anesthetized in 0.004% tricaine (ethyl 3-aminobenzoate methanesulfonate, MS-222, Sigma-Aldrich, St. Louis, MO, USA) and subjected to fin clip for a length of 2 mm. The fins were homogenized using Biomasher II (Nippi, Tokyo, Japan) in 290 μL of Genome Lysis Buffer (10 mM Tris–Cl (pH 8.0), 10 mM EDTA, 150 mM NaCl, 0.1% SDS, 33 ng/mL Proteinase K) for 3 h at 56 °C with thorough mixing by gentle inversion. Equal amounts (300 μL) of phenol/chloroform/isoamyl alcohol (25:24:1, pH 7.9) (Nacalai Tesque, Kyoto, Japan) were used to purify genomic DNA. After isopropanol (Nacalai Tesque) precipitation, the genome was washed with 80% EtOH followed by resuspension in 5 mM Tris–HCl (pH 8.0). Further clean-up of genomic DNA was conducted using AMpure XP beads (Beckman Coulter, Brea, CA, USA) according to the manufacturer’s manual. The concentration of the genomic DNA was measured using Quantus Fluorometer (Promega, Madison, WI, USA) and adjusted to 20 ng/μL.

### WGS library preparation and sequencing

The genomic DNA was converted to a next-generation sequencing library with 5× WGS Fragmentation mix and Ligase Mix (Enzymatics, Beverly, MA, USA). A total of 1 μL of genomic DNA was mixed with 1 μL of 10× Fragmentation Buffer, 2 μL of WGS Fragmentation Mix and 6 μL of nuclease-free water. The following program was used for fragmentation: 32 °C for 6.5 min followed by 65 °C for 30 min and a hold at 4 °C. Immediately after fragmentation, 4 μL of 5× Ligation Buffer, 2 μL of WGS Ligase and 6 μL of nuclease-free water were added. The following program was used for ligation: 20 °C for 15 min followed by a hold at 4 °C. The libraries were purified twice using 0.8× volume of AMpure XP beads (Beckman Coulter) and eluted in 12 μL of 5 mM Tris–HCl (pH 8.0). The adapter for the ligation step was prepared by annealing 100 mM of C*A*C*TCTTTCCCTACACGACGCTCTTCCGA*T*C*T and /5Phos/G*A*T*CGGAAGAGCACACGTCTGAACTCCAGT*C*A*C (* signifies a phosphorylated bond,/ /5Phos/s/ signifies a phosphorylation) with the following program: 95 °C for 2 min, gradually cooled to 45 °C (0.1 °C/s), followed by 45 °C for 5 min. Library amplification and adding index sequences to each end of the library were conducted by PCR. To determine an optimal number of cycles for PCR amplification, real-time PCR was conducted with 3.5 μL of adapter-ligated DNA, 5 μL of KAPA HiFi HotStart ReadyMix (Kapa Biosystems, Wilmington, MA, USA), 0.5 μL of Evagreen Dye (Biotium, Fremont, CA, USA), 0.5 μL of 10 μM Fw_i5 primer (AATGATACGGCGACCACCGAGATCTACACXXXXXXXXACACTCTTTCCCTACACGACGC) and 0.5 μL of 10 μM Rev_i7 primer (CAAGCAGAAGACGGCATACGAGATXXXXXXXXGTGACTGGAGTTCAGACGTGT). XXXXXXXX shown in the primer is an index sequence for multiplex sequencing (Table S7). The following program was used for real-time PCR: 95 °C for 5 min, 30 cycles of 98 °C for 20 s, 60 °C for 15 s, and 72 °C for 40 s followed by SYBR detection using the QuantStudio5 Real-Time PCR System (Thermo Fisher Scientific, Waltham, MA, USA). We selected the cycle number at the early exponential phase of the amplification curve. Then, the index was added to each sample by amplification with the optimal PCR cycles in 4 μL of the adapter-ligated DNA, 5 μL of KAPA HiFi HotStart ReadyMix (Kapa Biosystems), 0.5 μL of 10 μM Fw_i5 primer and 0.5 μL of 10 μM Rev_i7 primer. The libraries were purified twice using AMPure XP beads and eluted in 5 mM Tris–HCl (pH 8.0). The concentrations of the library were measured using Quantus Fluorometer. Then, 150 bp paired-end sequencing with DNBSEQ-T7 (MGI Tech, Shenzhen, China) was conducted by BGI Genomics (Shenzhen, China).

### Mapping and variant calling

The genome sequence of *Danio rerio* (Danio_rerio.GRCz11.dna.primary_assembly.fa.gz) is available at https://ftp.ensembl.org/pub/release-109/fasta/danio_rerio/dna. The reference sequence file for mapping was created for these genomic data, excluding extra data other than chromosomes (Chr 1–25) and the mitochondrial genome sequence (Danio_rerio.GRCz11.dna.primary_assembly.fa.gz). After trimming reads and removing adapter sequences using fastp version 0.20.1^36,37^ with the default parameters except for “--detect_adapter_for_pe: Specify that the sample is paired-end”, the data were mapped to the assembly genome (Danio_rerio.GRCz11.dna.primary_assembly-only-chr.fa) with BWA-MEM version 0.7.17-r1188^38^. Potential PCR duplicates were marked using the Mark Duplicates tool in Genome Analysis Toolkit GATK version 4.4.0.0^39^. SNPs were detected by the Haplotype Caller tool in GATK using the default parameters except for “-mbq 20: Minimum base quality needed to consider a base for calling” and the Select Variants tool in GATK. VCF-merge in the VCFtools version 0.1.16 package^40^ was used to merge all vcf files. Base quality recalibration was performed using the Variant Filtration tool in GATK, and raw SNPs were filtered with the following parameters: QD < 2.0: Variant confidence normalized by unfiltered depth of variant samples; FS > 60.0: Strand bias estimated using Fisher's Exact Test; MQ < 60: Root Mean Square of the mapping quality of reads across all samples; MQRankSum < − 12.5: Rank Sum Test for mapping qualities of REF versus ALT reads; ReadPosRankSum < − 8.0: Root Mean Square of the mapping quality of reads across all samples; and DP < 10: Depth of informative coverage for each sample. To assess how amino acids are affected by SNPs, genetic variants at the amino acid level were annotated using SnpEff version 4.3t and GRCz11 109^41^. The gene annotations were classified by SnpSift version 4.3t^42^ (https://github.com/Hirata-lab-2023/Inbred_strain/snpscall.sh).

### Phylogenetic analysis

To reveal the phylogenetic relationships of diverse zebrafish lines, we constructed a phylogenetic tree in the following four steps. The merged vcf file was converted to a PHYLIP file using vcf2phylip version 2.0^43^. A Python 3 script ascbias.py available at https://github.com/btmartin721/raxml_ascbias was used to remove inverted sites from the PHYLIP file. The edited PHLIP files were evaluated with the bioconda package ModelTest-NG version 0.1.7^14,44^ to select the best-fit model of evolution for DNA alignments. ML phylogenetic tree construction was carried out with RAxML-NG version 1.2.0^15,45^ using the TVM model and 10,000 bootstrap replicates. iTOL version 5^46^ was used for final editing (https://github.com/Hirata-lab-2023/Inbred_strain/phy.sh).

### SNP analysis

Heterozygous and homozygous SNP analysis was performed with a homemade script (https://github.com/Hirata-lab-2023/Inbred_strain/anlysis.R) using the core tools of R 4.2.3 and the R package—ggplot2 version 3.4.2^47^, openxlsx version 4.2.5.2, patchwork version 1.1.2, ggsignif version 0.6.4, dplyr version 1.1.2, ggbreak version 0.1.1^48^, stringr version 1.5.0, UpSetR version 1.4.0^49^, reshape2 version 1.4.4^47^, and sets version 1.0-24^50^. The numbers of homozygous and heterozygous SNPs were counted in each individual, and the percentages per total genome nucleotides were calculated. The number of heterozygous SNPs in each 100 kb region of the genome was also counted. All figures were edited using Adobe Illustrator version 26.4.1.

### Statistical analysis

Data are presented as the mean ± standard deviation of at least three independent experiments. Statistical differences between the target comparison groups were determined using unpaired Student’s *t* test or Welch’s t test. The results of the statistical test are indicated as *P ≤ 0.05, **P ≤ 0.01, ***P ≤ 0.001 or ****P ≤ 0.0001. P ≤ 0.05 was considered statistically significant. Graphical presentations were made with the R package ggplot2^47^.

### Ethics approval

All animal experiments were approved by the Animal Care and Ethics Committees of the National Institute of Genetics (22–13, 23–12, 24–12, 25–12, 26–11, 27–12, 28–13, 29–13, 30–14, 31–18, R2-8, R3-16 and R4-20), Keio University (14033, 17009, A2022-092) and Aoyama Gakuin University (A13) and carried out according to the Animal Research Reporting of In Vivo Experiments (ARRIVE) guidelines and to relevant guidelines and regulations.

### Supplementary Information

Supplementary Figures.

Supplementary Tables.

## Data Availability

Whole-genome sequences of AB (AB_SRA: SRR13015634) and TU (TU_SRA: ERR1992772) were obtained from the NCBI Sequence Read Archive. The reference TU genome (TU_GRCz11) was obtained from the Genome Reference Consortium^13,51^. Our sequencing data are deposited in the NCBI Sequence Read Archive as follows (Bio Project: PRJNA1002090): M-AB26_1: SRR25514300; M-AB26_2: SRR25514299; M-AB26_3: SRR25514298; *AB_1: SRR25514325; *AB_2: SRR25514324; *AB_3: SRR25514323; IM47_1: SRR25514304; IM47_2: SRR25514303; IM47_3: SRR25514302; IND_1: SRR25514329; IND_2: SRR25514328; IND_3: SRR25514327.
