# Supplementary material for: Establishment of a zebrafish inbred strain, M-AB, capable of regular breeding and genetic manipulation
Source: Sci Rep. 2024 Mar 29;14:7455. doi: 10.1038/s41598-024-57699-3 (PMC10978973; doi:10.1038/s41598-024-57699-3)
Supplement: Supplementary file 1 — Supplementary Figures. [file 41598_2024_57699_MOESM1_ESM.pdf]

**Supplementary Information**

**Table S1.** Fertility record of the IM strain at the 15th to 27th generations.

**Table S2.** Number of spawned eggs and fertilized eggs of the IM strain from the 46th to 49th generations.

**Table S3.** Number of heterozygous nucleotides for every 100 kb in chromosomes 1 to 25.

**Table S4.** List of genes disrupted in the M-AB strain.

**Table S5.** List of genes disrupted in the IM strain.

**Table S6.** List of genes disrupted in both the M-AB and IM strains.

**Table S7.** Index sequences used for genome sequencing.

A

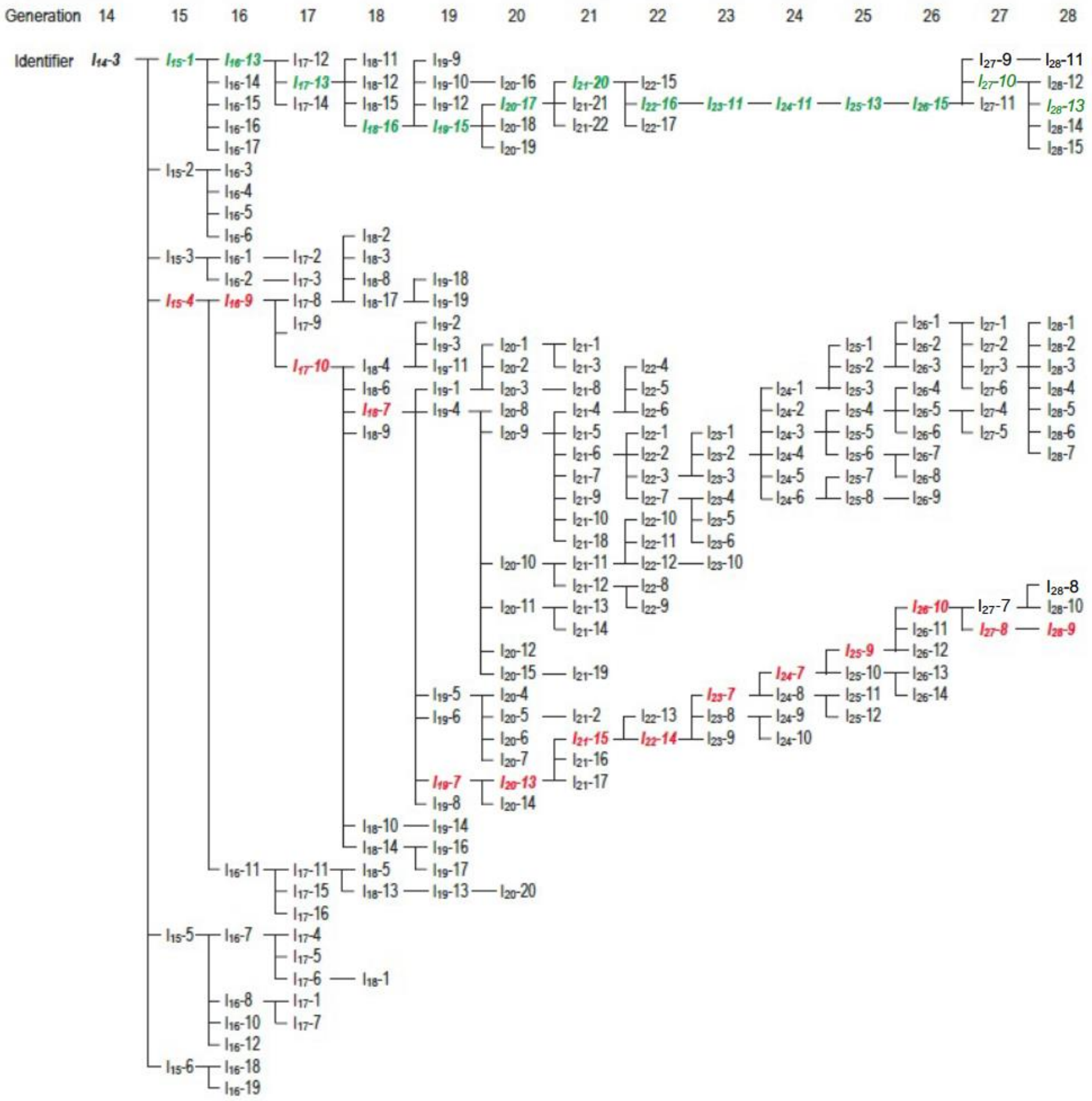
Generation 14 15 16 17 18 19 20 21 22 23 24 25 26 27 28
Identifier
$I_{14}$-3
$I_{15}$-1 $I_{15}$-2 $I_{15}$-3 $I_{15}$-4 $I_{15}$-5 $I_{15}$-6
$I_{16}$-13 $I_{16}$-14 $I_{16}$-15 $I_{16}$-16 $I_{16}$-17 $I_{16}$-3 $I_{16}$-4 $I_{16}$-5 $I_{16}$-6 $I_{16}$-1 $I_{16}$-2 $I_{16}$-9 $I_{16}$-11 $I_{16}$-7 $I_{16}$-8 $I_{16}$-10 $I_{16}$-12 $I_{16}$-18 $I_{16}$-19
$I_{17}$-12 $I_{17}$-13 $I_{17}$-14 $I_{17}$-2 $I_{17}$-3 $I_{17}$-8 $I_{17}$-9 $I_{17}$-10 $I_{17}$-11 $I_{17}$-15 $I_{17}$-16 $I_{17}$-4 $I_{17}$-5 $I_{17}$-6 $I_{17}$-1 $I_{17}$-7
$I_{18}$-11 $I_{18}$-12 $I_{18}$-15 $I_{18}$-16 $I_{18}$-2 $I_{18}$-3 $I_{18}$-8 $I_{18}$-17 $I_{18}$-4 $I_{18}$-6 $I_{18}$-7 $I_{18}$-9 $I_{18}$-10 $I_{18}$-14 $I_{18}$-5 $I_{18}$-13 $I_{18}$-1
$I_{19}$-9 $I_{19}$-10 $I_{19}$-12 $I_{19}$-15 $I_{19}$-18 $I_{19}$-19 $I_{19}$-2 $I_{19}$-3 $I_{19}$-11 $I_{19}$-1 $I_{19}$-4 $I_{19}$-5 $I_{19}$-6 $I_{19}$-7 $I_{19}$-8 $I_{19}$-14 $I_{19}$-16 $I_{19}$-17 $I_{19}$-13
$I_{20}$-16 $I_{20}$-17 $I_{20}$-18 $I_{20}$-19 $I_{20}$-1 $I_{20}$-2 $I_{20}$-3 $I_{20}$-8 $I_{20}$-9 $I_{20}$-10 $I_{20}$-11 $I_{20}$-12 $I_{20}$-15 $I_{20}$-4 $I_{20}$-5 $I_{20}$-6 $I_{20}$-7 $I_{20}$-13 $I_{20}$-14 $I_{20}$-20
$I_{21}$-20 $I_{21}$-21 $I_{21}$-22 $I_{21}$-1 $I_{21}$-3 $I_{21}$-8 $I_{21}$-4 $I_{21}$-5 $I_{21}$-6 $I_{21}$-7 $I_{21}$-9 $I_{21}$-10 $I_{21}$-18 $I_{21}$-11 $I_{21}$-12 $I_{21}$-13 $I_{21}$-14 $I_{21}$-19 $I_{21}$-2 $I_{21}$-15 $I_{21}$-16 $I_{21}$-17
$I_{22}$-15 $I_{22}$-16 $I_{22}$-17 $I_{22}$-4 $I_{22}$-5 $I_{22}$-6 $I_{22}$-1 $I_{22}$-2 $I_{22}$-3 $I_{22}$-7 $I_{22}$-10 $I_{22}$-11 $I_{22}$-12 $I_{22}$-8 $I_{22}$-9 $I_{22}$-13 $I_{22}$-14
$I_{23}$-11 $I_{23}$-1 $I_{23}$-2 $I_{23}$-3 $I_{23}$-4 $I_{23}$-5 $I_{23}$-6 $I_{23}$-10 $I_{23}$-7 $I_{23}$-8 $I_{23}$-9
$I_{24}$-11 $I_{24}$-1 $I_{24}$-2 $I_{24}$-3 $I_{24}$-4 $I_{24}$-5 $I_{24}$-6 $I_{24}$-7 $I_{24}$-8 $I_{24}$-9 $I_{24}$-10
$I_{25}$-13 $I_{25}$-1 $I_{25}$-2 $I_{25}$-3 $I_{25}$-4 $I_{25}$-5 $I_{25}$-6 $I_{25}$-7 $I_{25}$-8 $I_{25}$-9 $I_{25}$-10 $I_{25}$-11 $I_{25}$-12
$I_{26}$-15 $I_{26}$-1 $I_{26}$-2 $I_{26}$-3 $I_{26}$-4 $I_{26}$-5 $I_{26}$-6 $I_{26}$-7 $I_{26}$-8 $I_{26}$-9 $I_{26}$-10 $I_{26}$-11 $I_{26}$-12 $I_{26}$-13 $I_{26}$-14
$I_{27}$-9 $I_{27}$-10 $I_{27}$-11 $I_{27}$-1 $I_{27}$-2 $I_{27}$-3 $I_{27}$-6 $I_{27}$-4 $I_{27}$-5 $I_{27}$-7 $I_{27}$-8
$I_{28}$-11 $I_{28}$-12 $I_{28}$-13 $I_{28}$-14 $I_{28}$-15 $I_{28}$-1 $I_{28}$-2 $I_{28}$-3 $I_{28}$-4 $I_{28}$-5 $I_{28}$-6 $I_{28}$-7 $I_{28}$-8 $I_{28}$-10 $I_{28}$-9

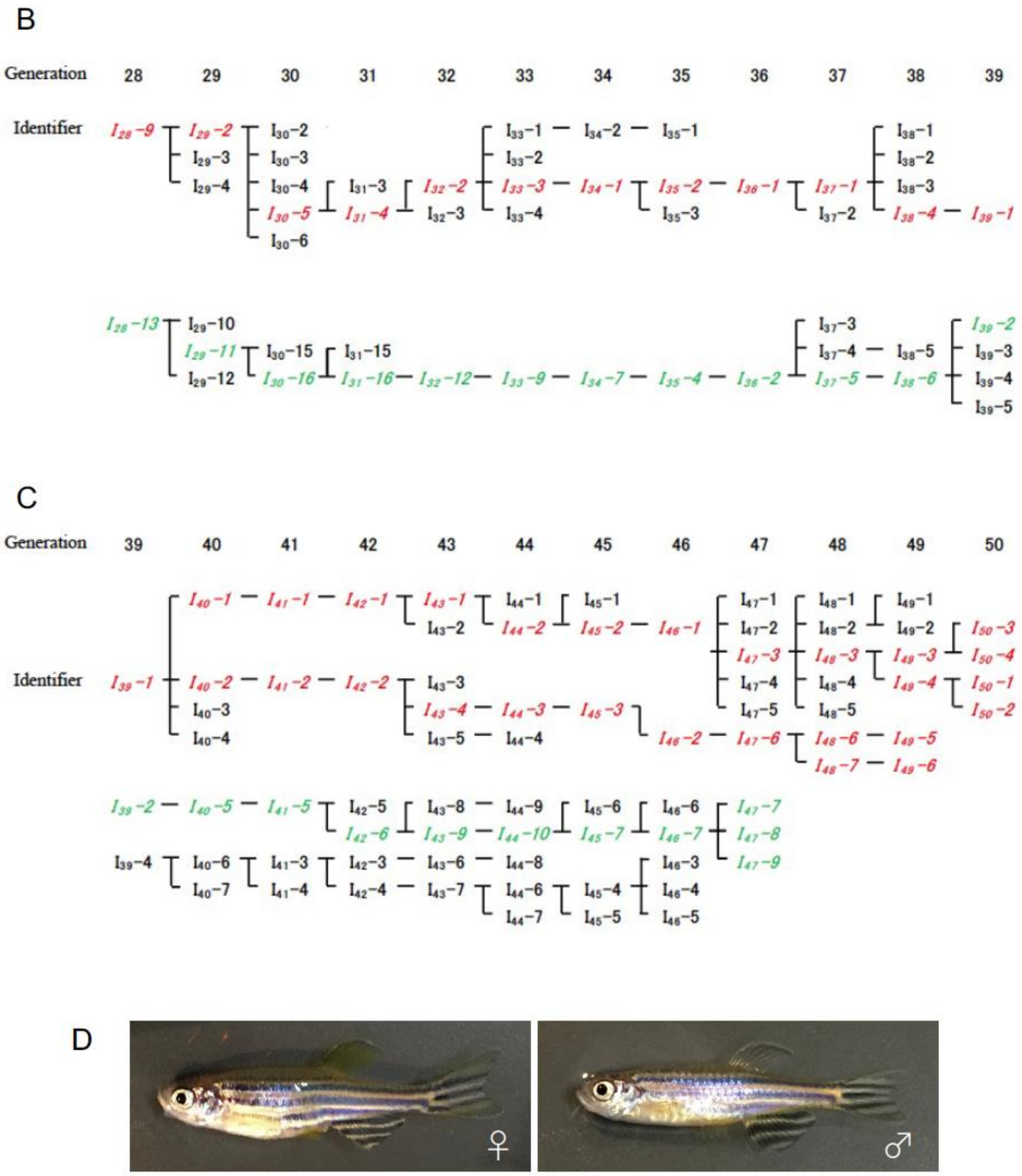


**Figure S1.** Pedigree of the IM strain. **(**A) Pedigree from generation 14 to 28. (B) Pedigree from generation 28 to 39. (C) Pedigree from generation 39 to 48. Pairs in italics indicate pairs that are connected to existing pairs. There were two subfamilies (green and red). (D) Females and males of the M-AB strain.

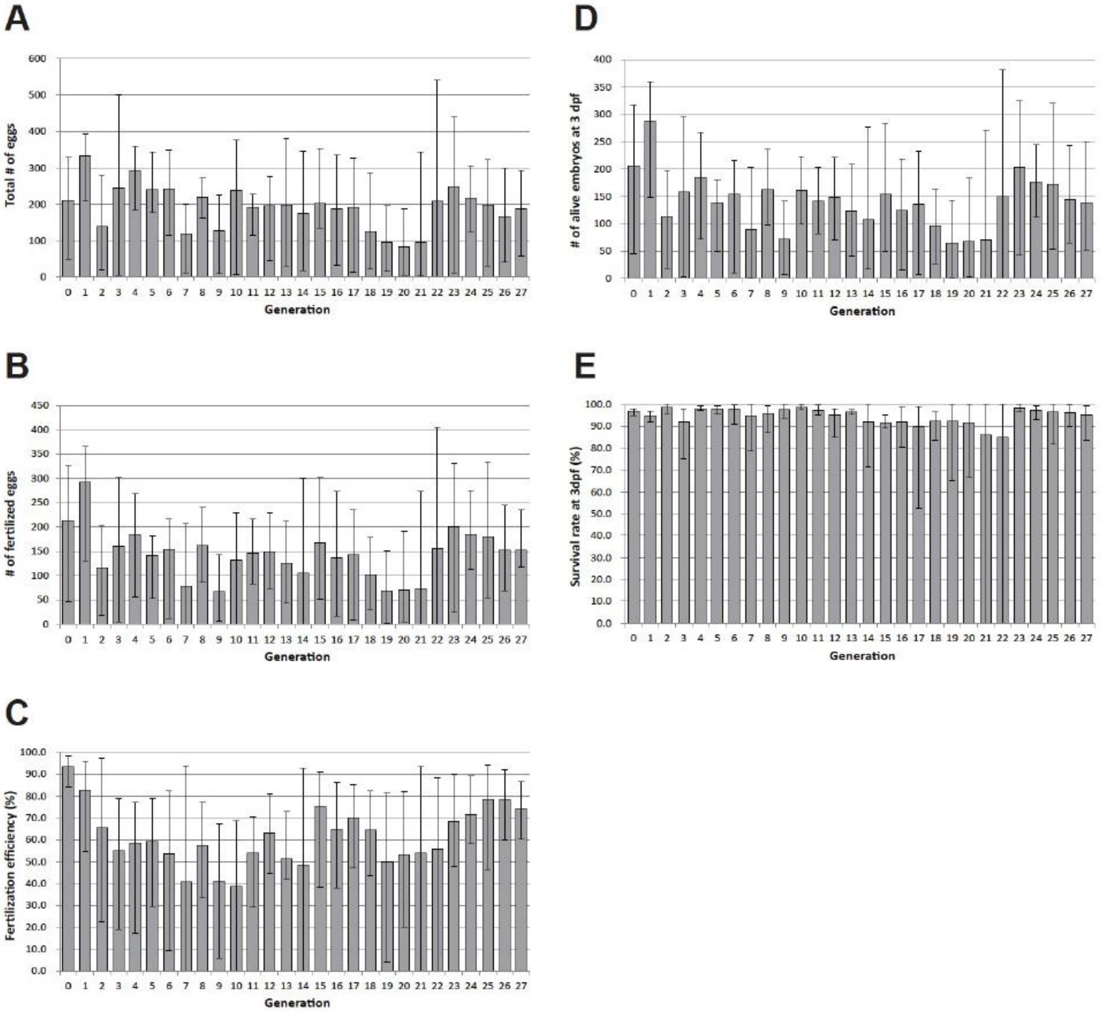


**Figure S2.** Inbreeding depression of the IM strain. (A) Number of spawned eggs per clutch. (B) Number of fertilized eggs per clutch. (C) Percentage of fertilized eggs per clutch. (D) Number of embryos that survived up to 3 dpf per clutch. (E) Percentage of embryos that survived up to 3 dpf per clutch. Error bars indicate the maximum (highest) and minimum (lowest) values. Data from generations 0 to 15 were shown in a previous report [11].

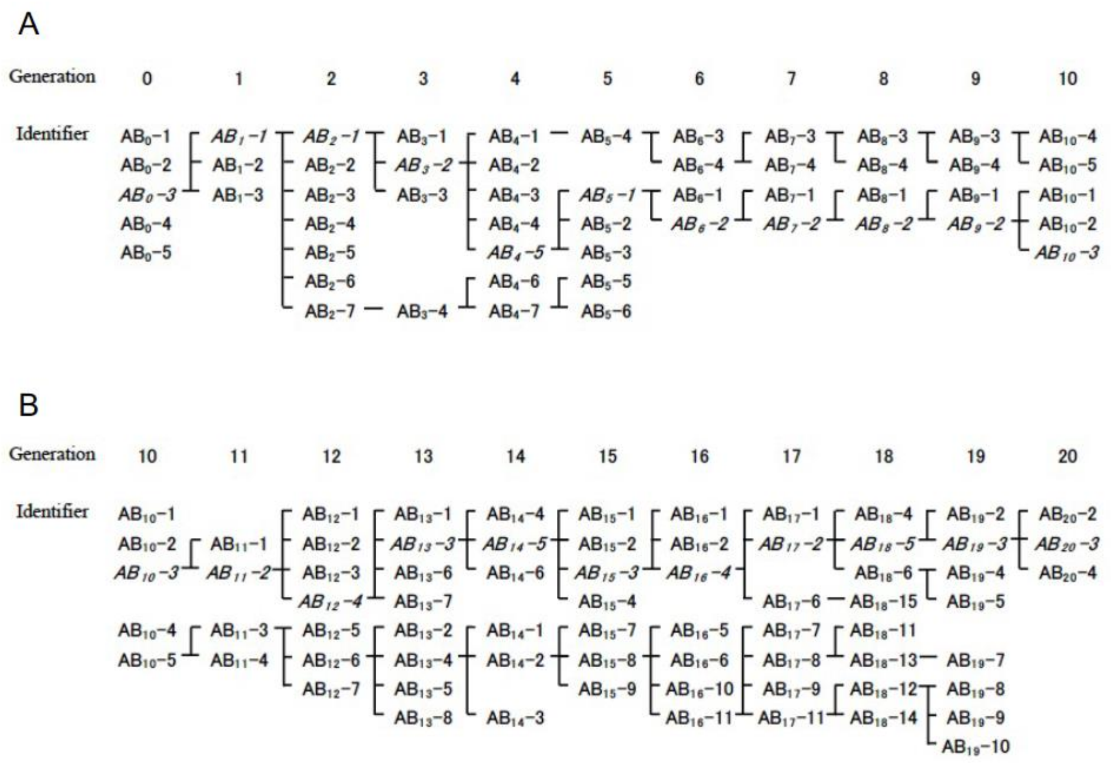


**Figure S3.** Pedigree of the M-AB strain. (A) Pedigree from generation 0 to 11. (B) Pedigree from generation 11 to 20. Pairs in italics indicate pairs that are connected to existing pairs.

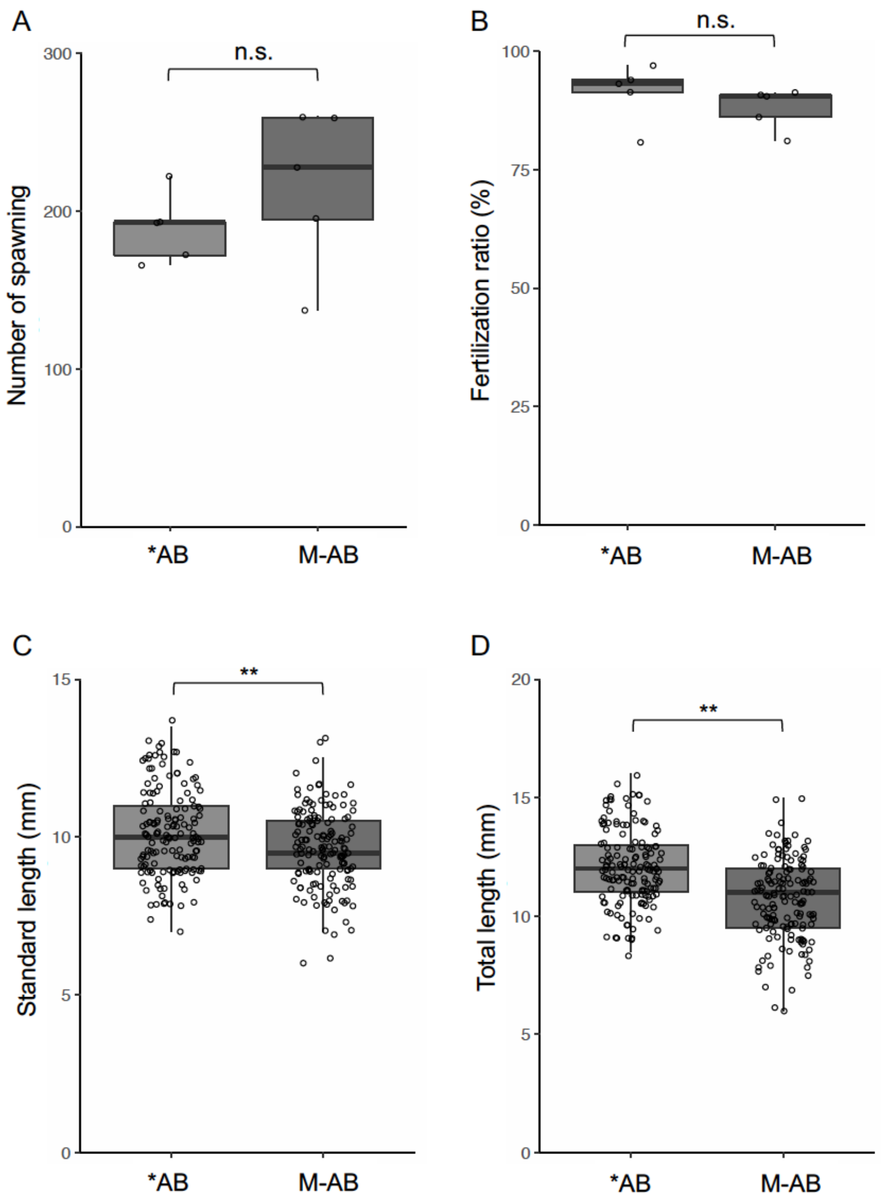


**Figure S4.** Fertility and growth of the M-AB and *AB fish. **(**A) The number of fertilized eggs of M-AB ($AB_{27}$-5) and *AB (N = 5). (B) The efficiency of fertilization of M-AB ($AB_{27}$-5) and *AB (N = 5). (C) Standard length (from head to the root of the caudal fin) of M-AB and *AB fish at 30 dpf. (D) Total length (from head to the end of caudal fin) of M-AB and *AB fish at 30 dpf. For standard and total length, 30 larvae were randomly selected for measurement with 5 duplicates each. The data are presented as boxplot diagrams. P values were calculated using unpaired Student's t test, **$P \leq 0.01$, n.s., not significant.

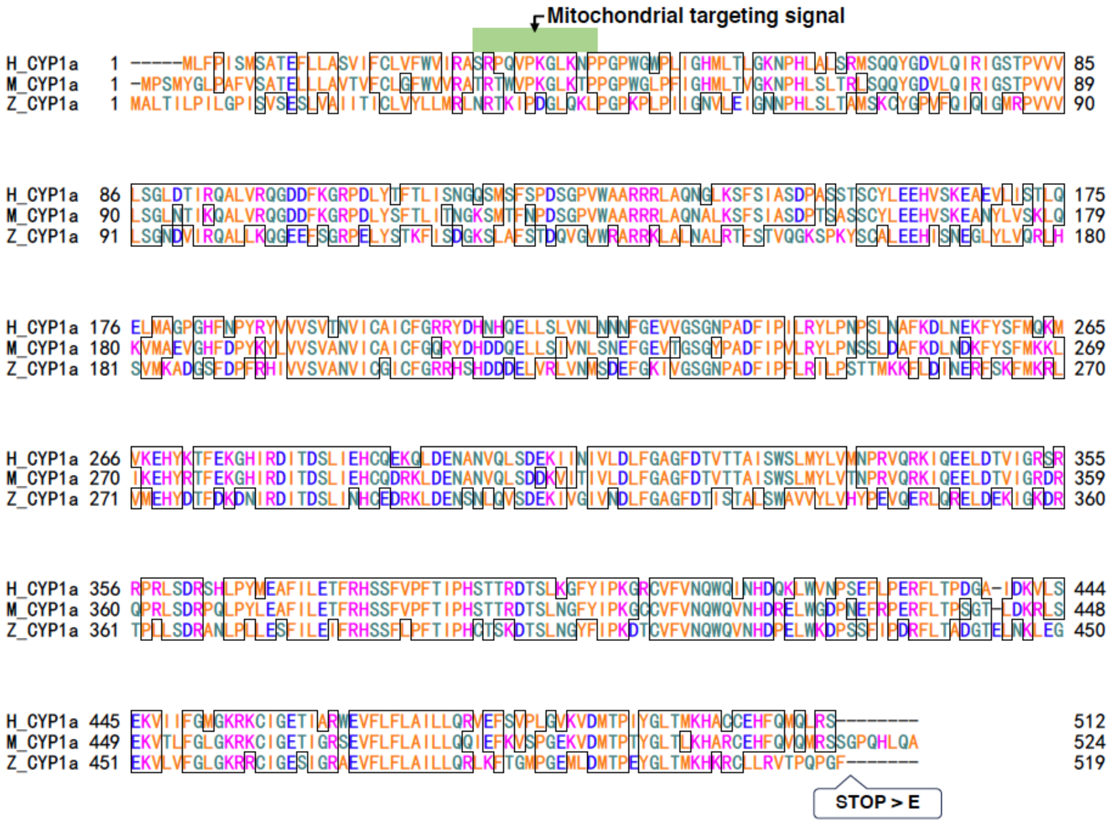


**Figure S5.** A SNP in the *cyp1a1* gene, which was found in the IM strains, causes synthesis of C-terminally extended protein. Amino acid alignment of Cyp1a1 protein from humans (NP_000490.1), mice (NP_001129531.1) and zebrafish (NP_571954.1). A SNP in the zebrafish IM strain disrupts the termination codon, resulting in the X520Glu4X variant.

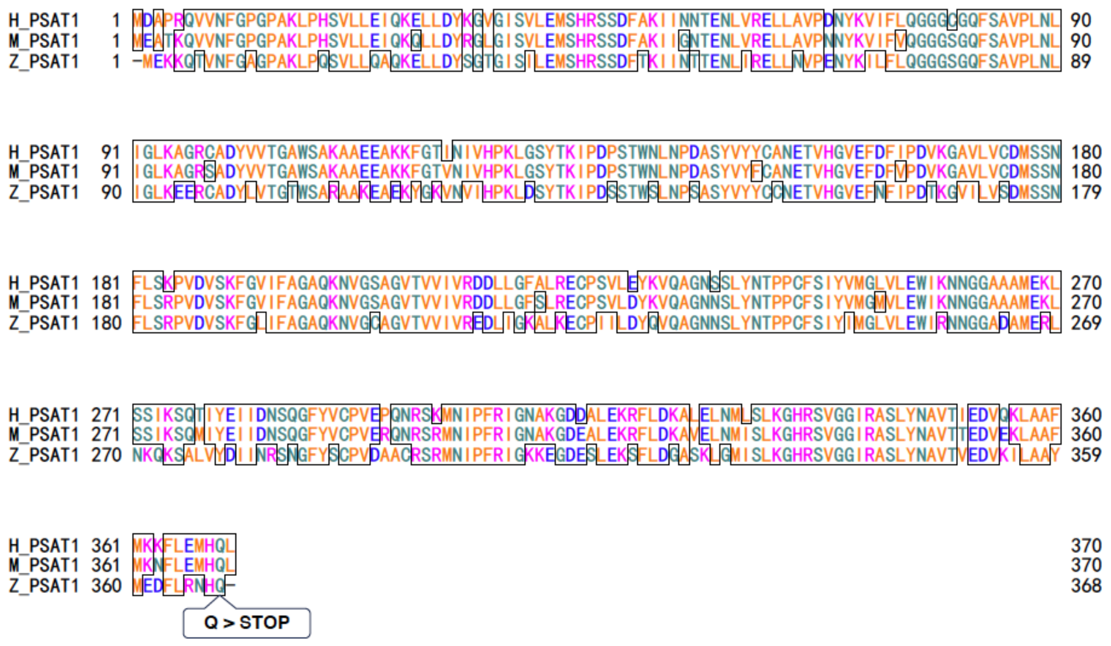


**Figure S6.** A SNP in the *psat1* gene, which was found in the IM strains, causes truncation of the protein. Amino acid alignment of Psat1 protein from humans (NP_478059.1), mice (NP_803155.1) and zebrafish (NP_956113.1). A SNP in the zebrafish IM strain generates a nonsense codon just before the termination codon, resulting in the Q368X variant.

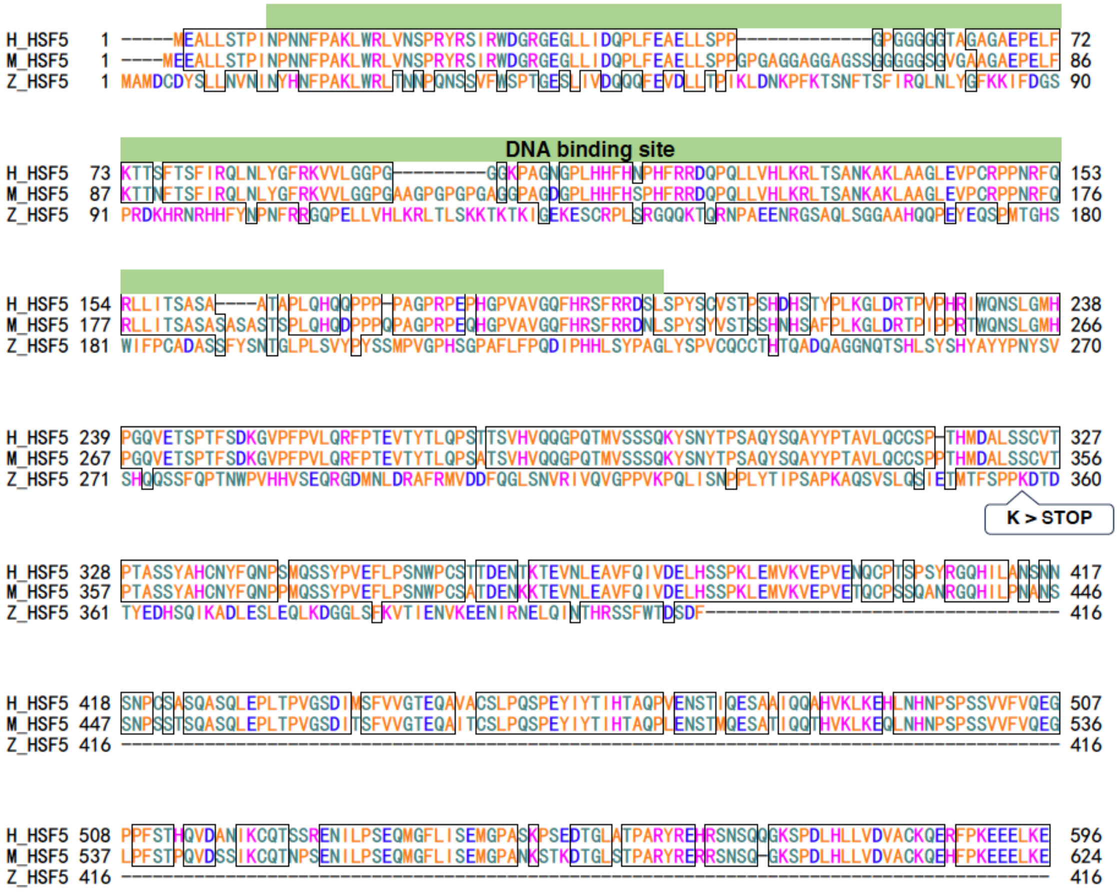


**Figure S7.** SNP in the *hsf5* gene, which was found in the IM strain, causes truncation of the protein. Amino acid alignment of Hsf5 protein from humans (NP_001073908.2), mice (NP_001038992.1) and zebrafish (NP_001082945.1). A SNP in the zebrafish IM strain generates a nonsense codon, resulting in the K357X variant.

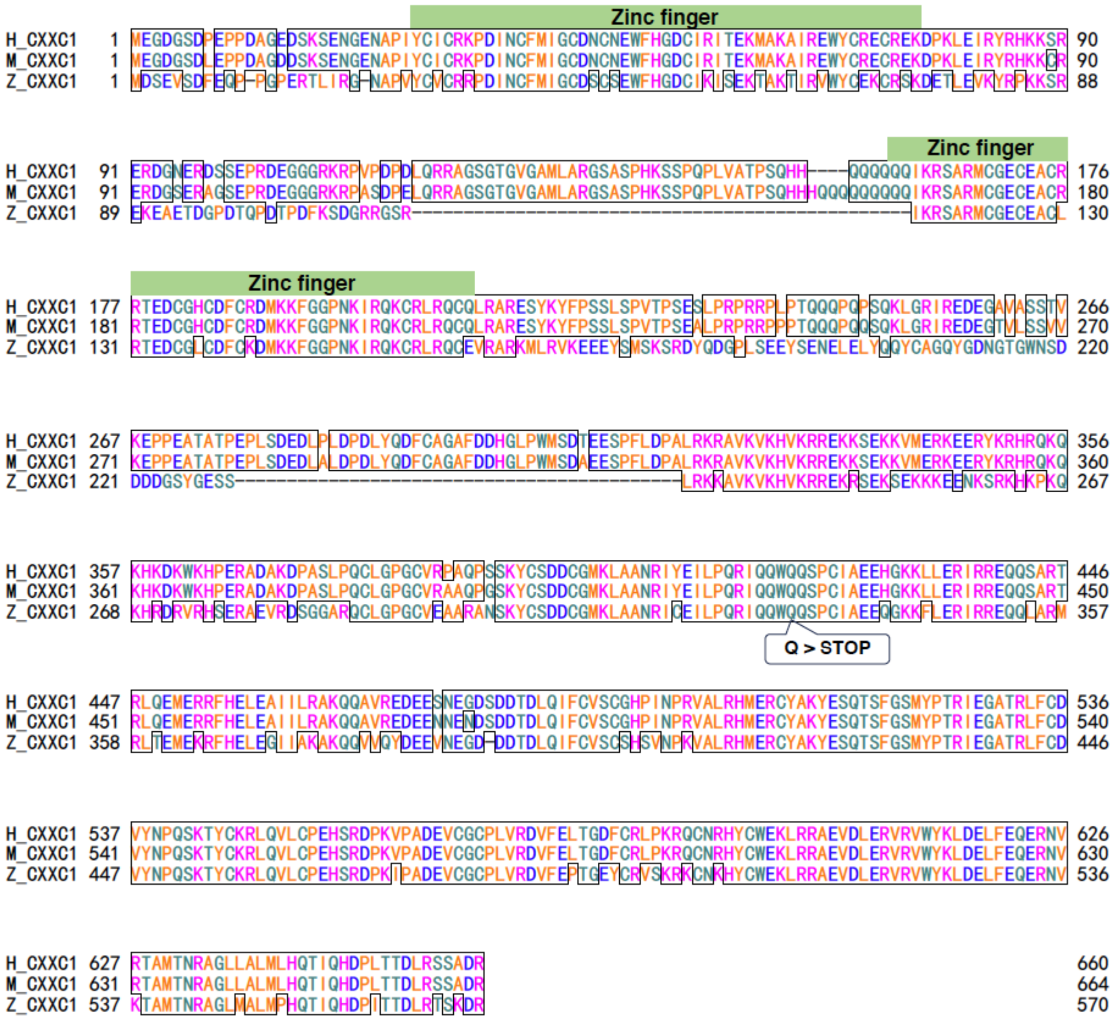

**Figure S8.** A SNP in the *cxxc1* gene, which was found in the IM strains, causes truncation of the protein. Amino acid alignment of CXXC1 protein from humans (NP_001095124.1), mice (NP_001365773.1) and zebrafish (NP_956893.1). A SNP in the zebrafish IM strain generates a nonsense codon, resulting in the Q331X variant.

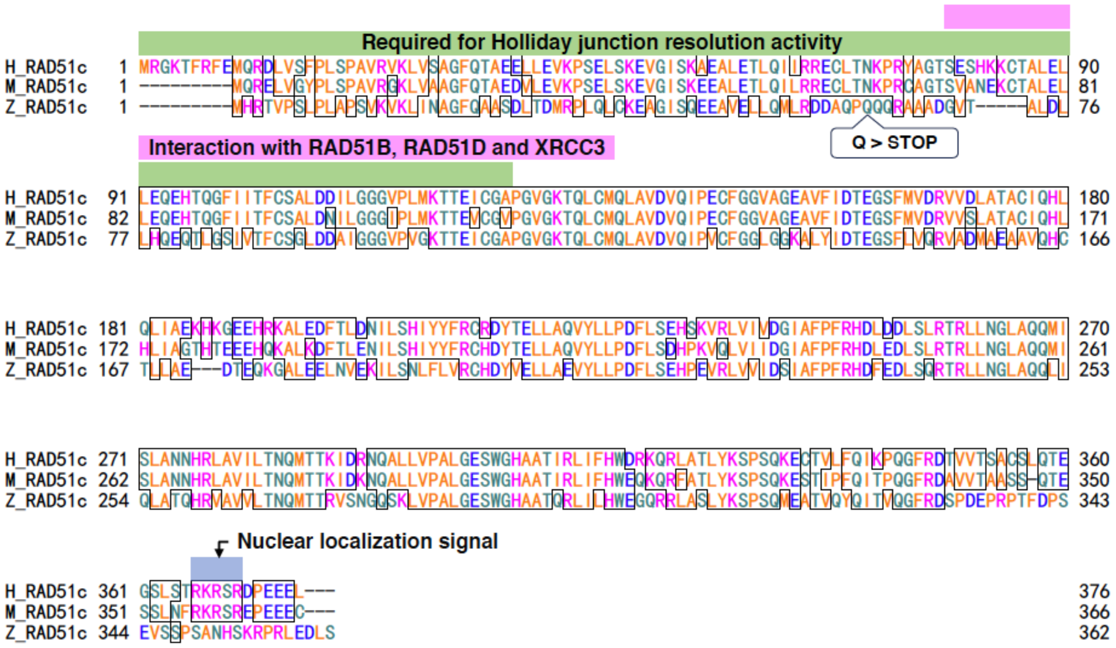


**Figure S9.** A SNP in the *rad51c* gene, which was found in the IM strains, causes truncation of the protein. Amino acid alignment of Rad51c protein from humans (NP_478123.1), mice (NP_444499.1) and zebrafish (NP_001006101.1). A SNP in the zebrafish IM strain generates a nonsense codon, resulting in the Q62X variant.

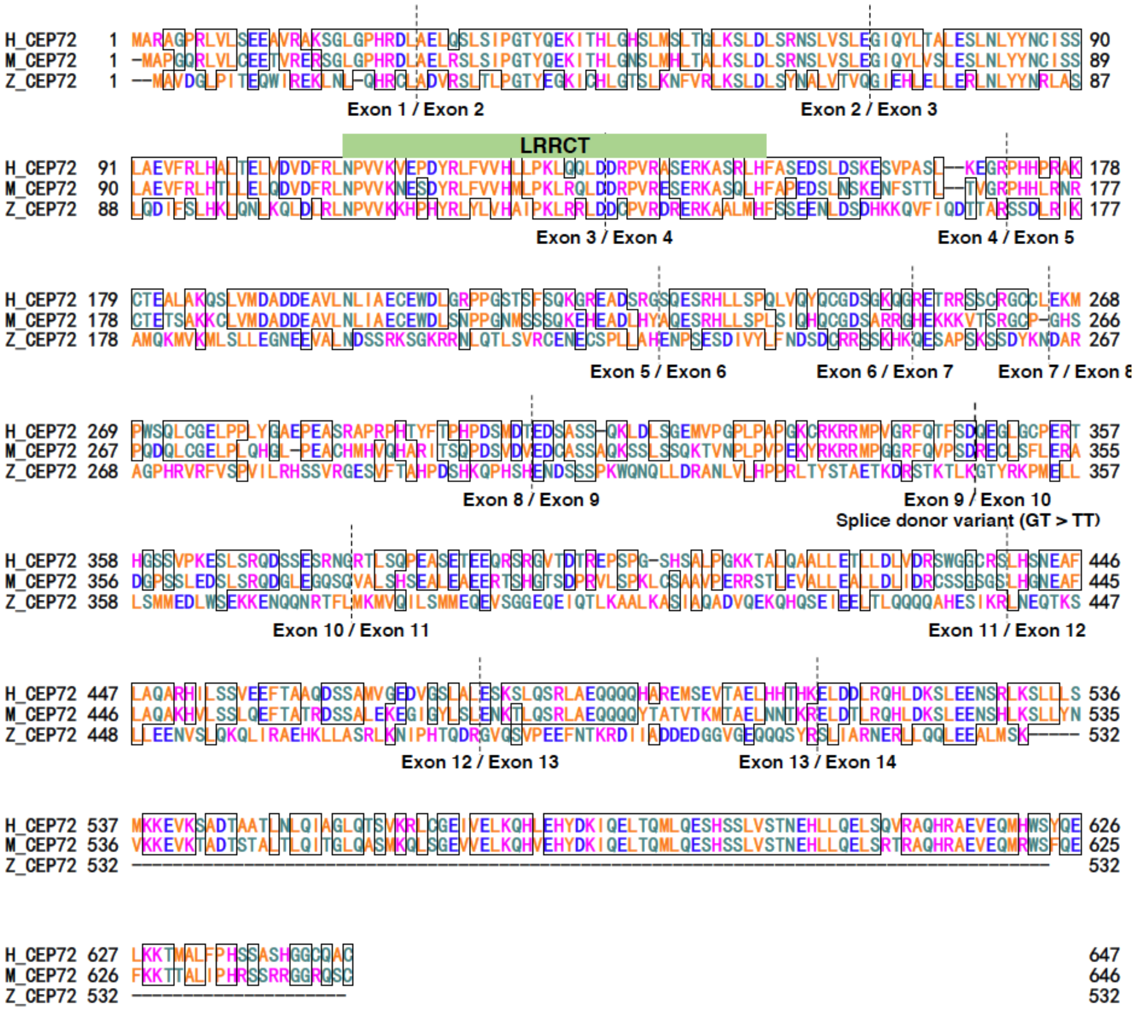

**Figure S10.** A SNP in the *cep72* gene, which was found in the IM strains, should affect splicing of the transcripts. Amino acid alignment of Cep72 protein from humans (NP_060610.2), mice (NP_083235.3) and zebrafish (NP_001306061.1). A SNP in the zebrafish IM strain disrupts the splice donor site.

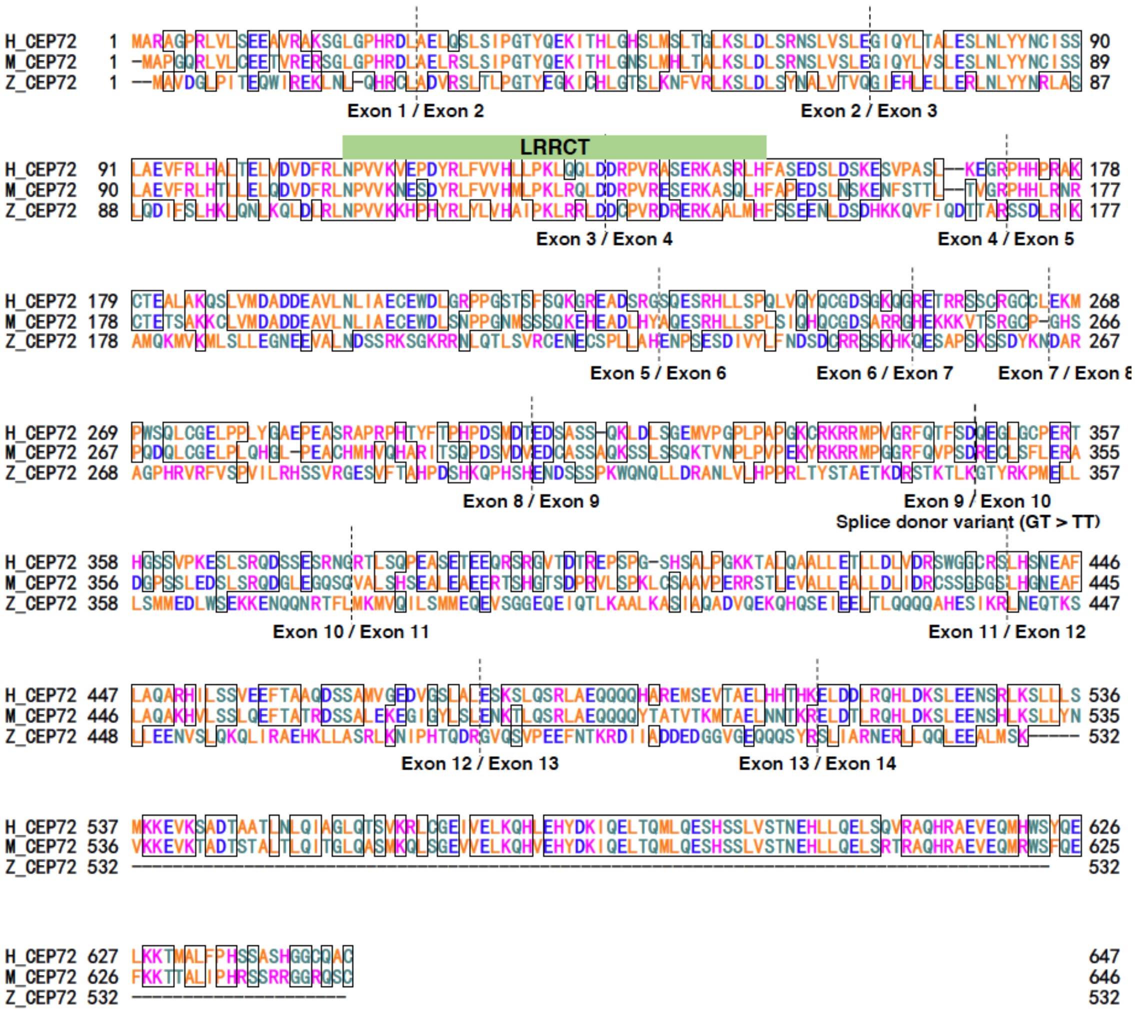


**Figure S11.** A SNP in the *fbxo42* gene, which was found in the IM strains, causes truncation of the protein. Amino acid alignment of Fbxo42 protein from humans (NP_061867.1), mice (NP_766106.2) and zebrafish (NP_766106.2). A SNP in the zebrafish IM strain generates a nonsense codon, resulting in the R297X variant.

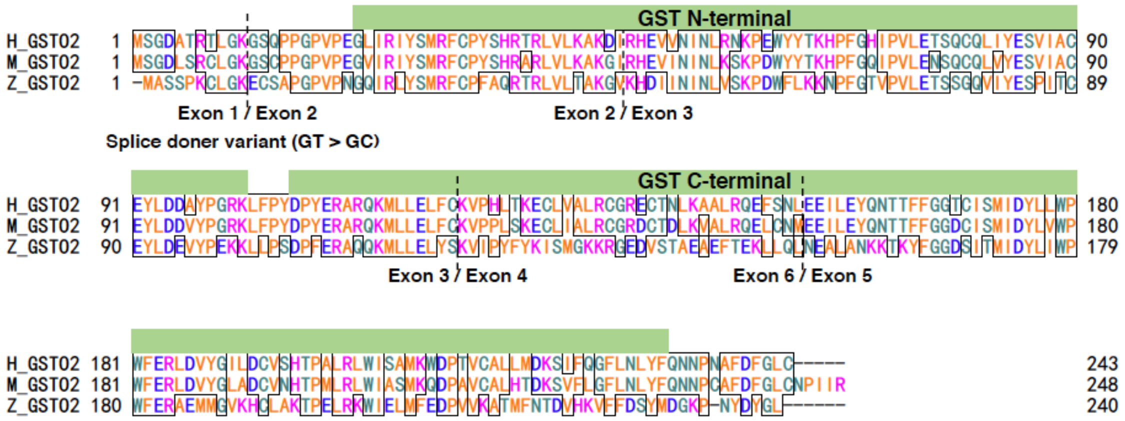


**Figure S12.** A SNP in the *gsto2* gene, which was found in the IM strains, should affect splicing of the transcripts. Amino acid alignment of Gsto2 protein from humans (NP_899062.1), mice (NP_080895.2) and zebrafish (NP_001007373.1). A SNP in the zebrafish IM strain disrupts the splice donor site.

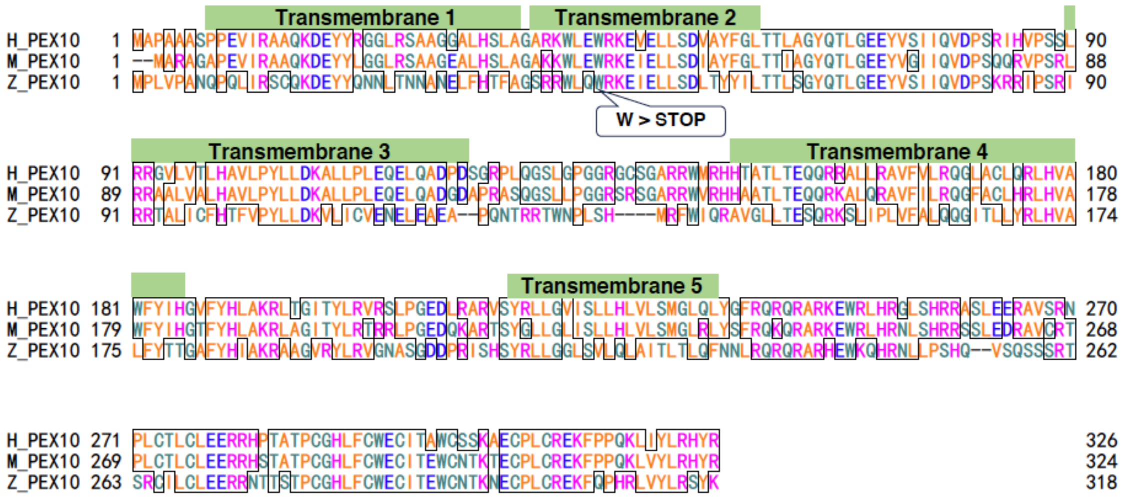


**Figure S13**. A SNP in the *pex10* gene, which was found in the IM strains, causes truncation of the protein. Amino acid alignment of Pex10 protein from humans (NP_002608.1), mice (NP_001035866.1) and zebrafish (NP_001005994.1). A SNP in the zebrafish IM strain generates a nonsense codon, resulting in the W45X variant.

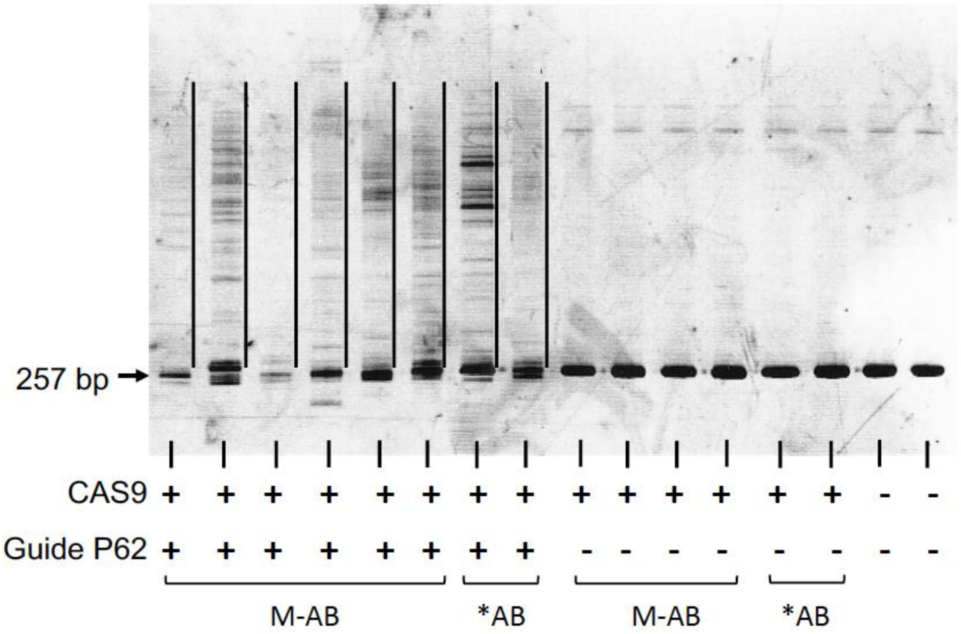


**Figure S14.** Heteroduplex mobility assay of the *sqstm1* gene. The genome for PCR was extracted from embryos (1 dpf) injected with or without the sgRNA/Cas9 mix or Cas9 alone. The position of the expected homoduplexes is indicated by the arrow, and the multiple heteroduplexes are indicated by the vertical lines.

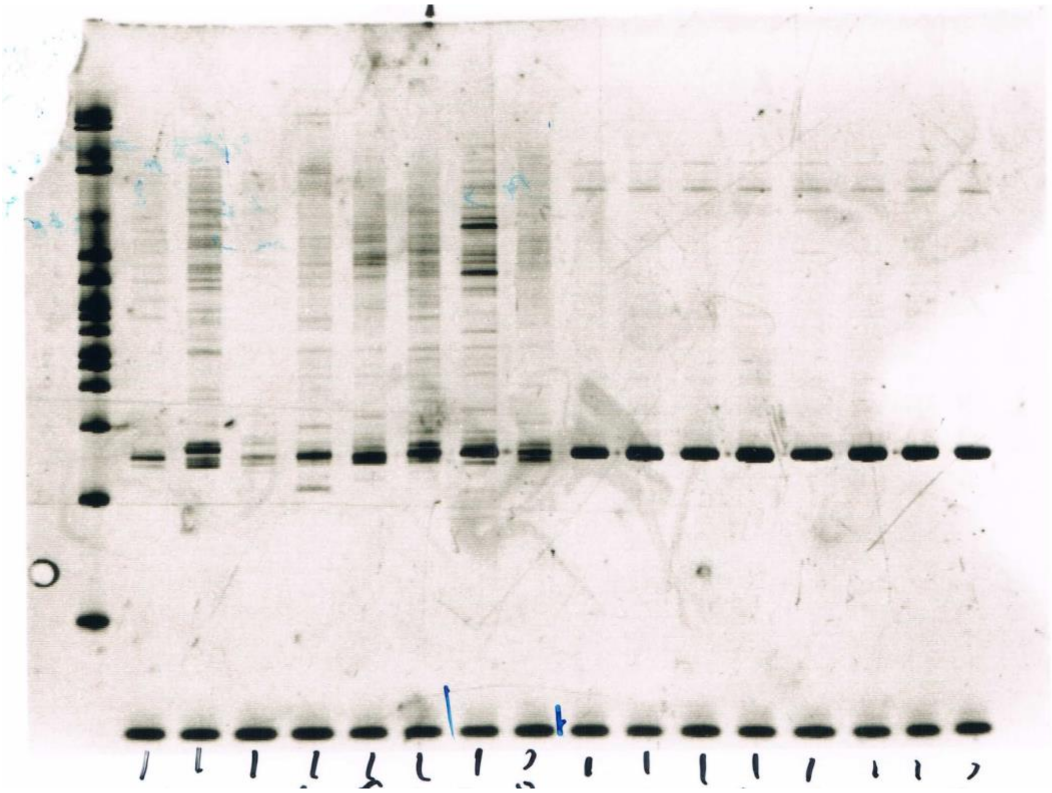

**Figure S15.** The image of figure S14 showing full length gel with edges.
